# Supplementary material for: Parametrically guided design of beta barrels and transmembrane nanopores using deep learning
Source: Proc Natl Acad Sci U S A. 2025 Sep 15;122(38):e2425459122. doi: 10.1073/pnas.2425459122 (PMC12478100; doi:10.1073/pnas.2425459122)
Supplement: Supplementary file 1 [file pnas.2425459122.sapp.pdf]

PNAS

Supporting Information for

Parametrically guided design of soluble beta barrels and transmembrane nanopores using deep learning

David E. Kim, Joseph L. Watson, David Juergens, Sagardip Majumder, Ria Sonigra, Stacey R. Gerben, Alex Kang, Asim K. Bera, Xinting Li, and David Baker

David Kim
Email: dekim@uw.edu

This PDF file includes:

Supporting text
Figures S1 to S8
Tables S1 to S3
SI References

Other supporting materials for this manuscript include the following:

RFjoint2 code and model weights are available at:

https://github.com/RosettaCommons/RFjoint2

RFdiffusion code and model weights are available at:

https://github.com/RosettaCommons/RFdiffusion

Utility scripts to generate input barrel backbones, RFjoint2 and RFdiffusion refinement commands, and to determine the extent of barrel closure are available at:

https://github.com/davidekim/parametric_barrels

Analysis scripts for processing ion conductance data are available on Github and archived in Zenodo.

https://github.com/sagardipm/denovoPores
DOI: 10.5281/zenodo.10939541

Supporting text

RFjoint2

We explored whether we could use RoseTTAFold based inpainting (1) for the design of beta barrels starting from global geometric descriptions. We trained an improved version of RFjoint, RFjoint2, which outperforms the original RFjoint model at motif scaffolding (Fig. S1) and has not been described elsewhere. Several key improvements underpin RFjoint2 and are described in the methods section below. Firstly, RFjoint2 trains from a later version of RoseTTAFold, which was the first RoseTTAFold version to explicitly model sidechains (the original RF and RFjoint models only predicted backbone atoms) parameterized by torsion angles (2). Secondly, in contrast to RFjoint, where sequence was decoded in “one-shot” by taking the most probable amino acid at each position, sequence in RFjoint2 is decoded iteratively over many “recycles”.

On the benchmark set of motif-scaffolding problems introduced in the RFdiffusion manuscript (3), RFjoint2 sequences significantly improve over RFjoint sequences (higher success rates in 14 cases, and one tie) (Table S1). With ProteinMPNN sequence re-design of the designed scaffold, RFjoint2 “solves” 21 of the benchmarking problems (vs 23 in RFdiffusion and 17 in RFjoint), with higher in silico success rates than RFjoint in 15 of these cases. While RFjoint2 still generally performs worse than RFdiffusion, RFjoint2 outperforms RFdiffusion on 6 of the benchmarking problems (Table S1).

Supporting methods

RFjoint2 Training

RFjoint2 was trained from an unpublished developmental version of RoseTTAFold2 (4) (RF2). For clarity, we name this network RF-intermediate. This network is not a contribution of this work. For context, we provide a brief overview of the training of this network below.

RoseTTAFold Structure Prediction Training

The RF architecture used for RFjoint2 differs significantly from the original RF architecture (2). Notably, unlike the original RF model, RF-intermediate explicitly models amino acid sidechains, using the parameterization in AF2 and RF2 (4, 5). Unlike RF2 and AF2, RF-intermediate was trained only on the PDB (without an AF2 distillation set). Following AF2 (5), the frame-aligned point error (FAPE) loss is the primary structural loss used during training.

Hyperparameters are detailed below:

- Training set: PDB set only (RoseTTAFold training set), 21120 examples per epoch
- Optimizer: AdamW (6) optimizer with default parameters in PyTorch (7)
- Batch size: 64 examples per batch
- Learning rate: 0.0005

- Linear warm-up for 16000 optimization steps, followed by;
- Linear decay for 200000 optimization steps
- Crop size: 300 amino acids
- Number of seed MSA sequences: 128
- Number of extra MSA sequences: 2048
- 300 epochs of training

The losses used were the following, and are fully described in the RF2 manuscript (4).

- Distogram loss (L_{dist}); 1.0
- FAPE loss (L_{FAPE}); 10.0 (80:20 backbone only vs full-atom)
- LDDT loss (L_{lddt}); 0.1
- MSA prediction loss (L_{MLM}); 3.0

RFjoint2 Training Parameters

RFjoint2 was fine-tuned directly from the RF-intermediate weights, and in an otherwise identical manner to the original RFjoint model (1), using the same training tasks (structure prediction, fixed-backbone sequence prediction and joint sequence-structure inpainting) and masking strategy. RFjoint2 was trained using the same losses as RF-intermediate, except for the addition of a clash loss (L_{clash}), described elsewhere (4), with weight 1.0.

Training hyperparameters are detailed below:

- Three training tasks (as in RFjoint (1)); Structure prediction (as in RF), fixed-backbone sequence design, joint sequence structure "inpainting". Sampled with equal probability.
- During fixed-backbone sequence design training, 90-100% of residues were masked.
- During inpainting training, 10-35 contiguous residues were masked, flanked by 3-6 residues either side, where a loss was not applied (see RFjoint (1)).
- Training set: PDB set only (RoseTTAFold training set), 21120 examples per epoch
- Optimizer: AdamW (6) optimizer with default parameters in PyTorch (7)
- Batch size: 512 examples per batch
- Learning rate: 0.0005
- Linear warm-up for 16000 optimization steps, followed by;
- Linear decay for 200000 optimization steps
- Crop size: 260 amino acids
- 50 epochs of training

RFjoint2 Inference

While the training of RFjoint2 very closely matched the training of RFjoint, the inference strategy deviated significantly. The details of the changes are described below.

Recycling

RF, following AF2, uses "recycling" during structure prediction to iteratively refine a prediction (by re-running prediction with the latent prediction from the previous recycle). In RFjoint, recycling was also used, typically running 5 recycles during a prediction. However, in RFjoint2 we recycle for significantly longer. By applying the L_{lddt} loss during RFjoint2 training, we handily maintain a "confidence" parameter that we can use to monitor the progress of an RFjoint2 trajectory. Because this loss penalizes the model's assessment of how far the "inpainted" structure is from the true structure, the RFjoint2 pLDDT parameter correlates with structure quality. Through recycling, the pLDDT typically increases, and hence, we recycle until the pLDDT has plateaued (no improvement for 10 recycles). This often leads to in excess of 50 recycles. At this point, we begin autoregressive sequence design (see below).

Autoregressive sequence design

In RFjoint, the amino acid sequence was generated "one-shot", by taking the most probable amino acid predicted at each position. Inspired by the iterative decoding in ProteinMPNN (8), in RFjoint2, after the pLDDT has plateaued (see above), we iteratively "decode" sequence, by sampling from the softmaxed-logit probabilities at a random position at each recycle. This position then gets fixed and input as "true" sequence to the next recycle. Because sequence design is a locally-defined problem (8), we simultaneously decode multiple positions at a time (through a greedy search permitting decoding of residues > 15 Å to be simultaneously decoded). This leads to a significant inference speed up. For all experiments, the softmax temperature for sampling was 0.1, following the default in ProteinMPNN. The random decoding order also creates stochasticity in the outputs of RFjoint2, in comparison to the deterministic inference used in RFjoint. This order-agnostic autoregressive sequence design strategy nearly matches the quality of sequences generated with fixed-backbone sequence design using ProteinMPNN (8), but we found it most expedient to generate 8 ProteinMPNN sequences per RFjoint2-inpainted backbone (3, 9, 10), and choose the sequence with the closest recapitulation of the design model according to AlphaFold2 (AF2) (5) structure prediction.

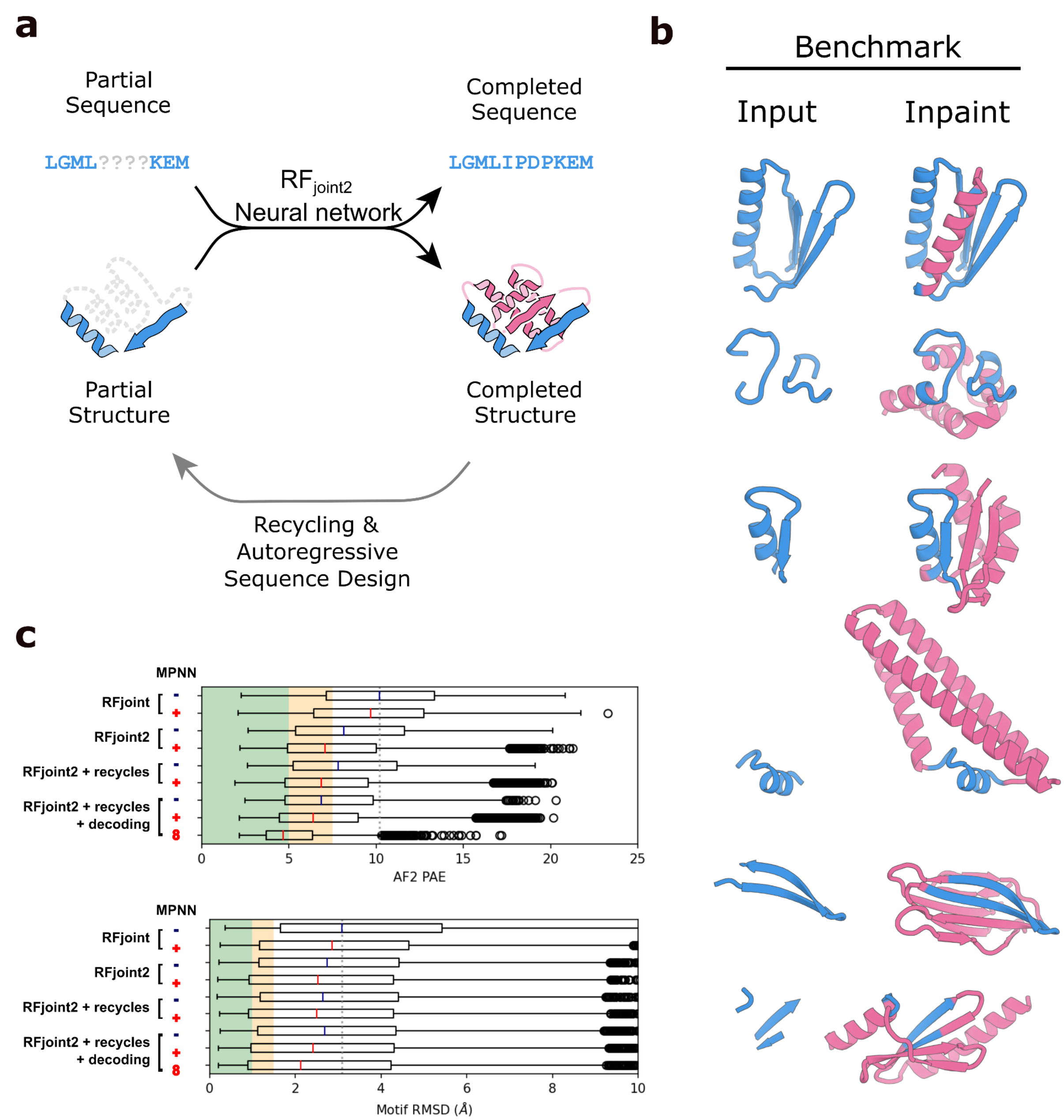

Fig. S1. Overview of RFjoint2 inpainting

a) Overview of RFjoint2. RFjoint2 takes as input the sequence and structure of a “motif” (blue). It then predicts the sequence and structure of the “scaffold” around the motif (pink). Predictions are made over several “recycles”, and the sequence is iteratively decoded in an order-agnostic manner. **b**) A few examples from the RFdiffusion motif-scaffolding benchmark set, where RFjoint2 significantly outperforms RFjoint (examples are *2KL8, 1PRW, 5TPN, 6E6R_long, 5IUS, 5YUI*). Full benchmarking details are listed in Table S1. **c**) Ablation experiment showing the effect of different features in RFjoint2, as evaluated by AF2 confidence (pAE, top) and recapitulation of the motif (bottom). RFjoint2 outperforms RFjoint, with and without

ProteinMPNN sequence redesign of the de novo scaffold. Permitting indefinite recycling in RFjoint2 (until pLDDT plateaus; see Methods) subtly improves RFjoint2 outputs. Furthermore, subsequent order-agnostic autoregressive sequence design improves both the sequences (blue) and backbones (with ProteinMPNN sequences; red) designed by RFjoint2. Sampling 8 sequences in ProteinMPNN and taking the best (in line with other work (3, 9, 10)), unsurprisingly, leads to improved sequences.

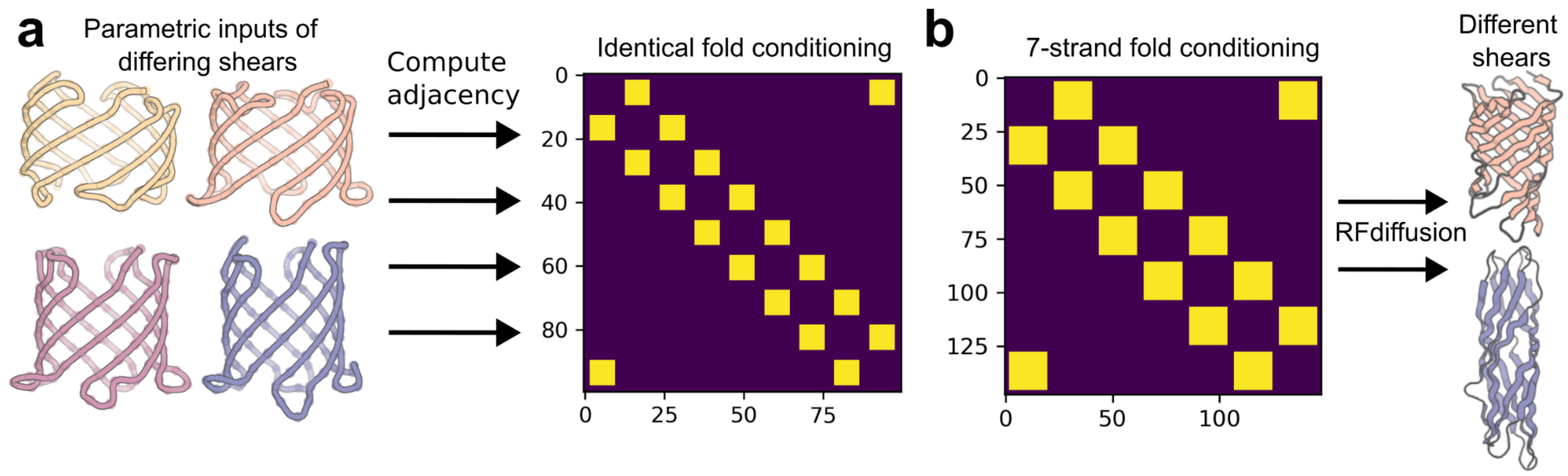

Fig. S2. RFdiffusion fold conditioning cannot perform parametric beta barrel design.

a) Using the published code for RFdiffusion to compute “block adjacency” (fold conditioning) matrices for four parametric beta barrel inputs (left) with identical parameters other than their strand shear yields the same block adjacency matrix (right). Thus suggesting the shear parameter is not encoded by the block adjacency featurization. **b**) When generating 150 outputs with RFdiffusion using a block adjacency matrix consistent with a seven-strand beta barrel topology (left), RFdiffusion generated barrels with inconsistent shear (right).

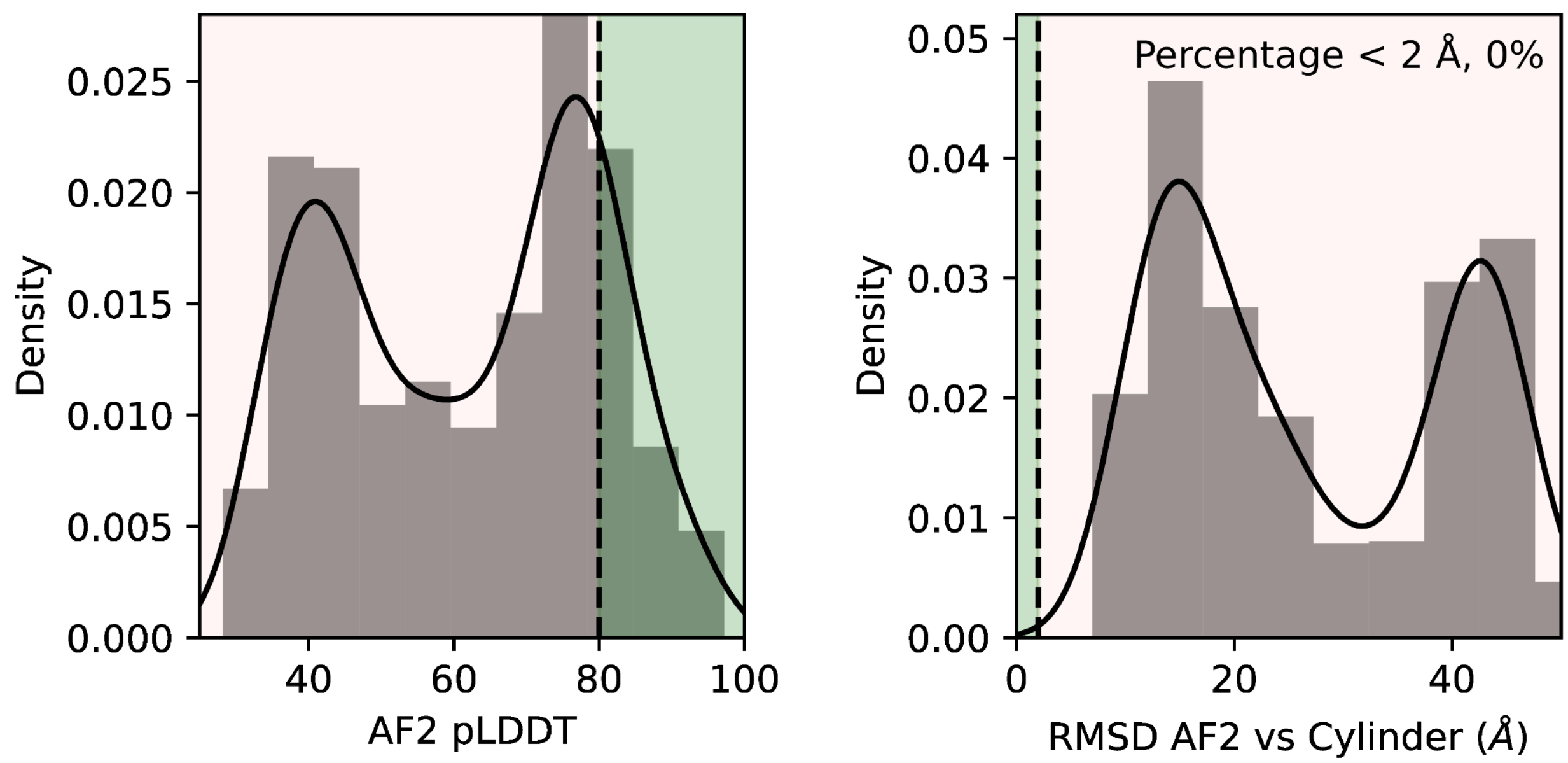

Fig. S3. Parametric cylinders are not designable.

Without RFdiffusion/RFjoint2 refinement, the parametric backbone cylinders (with loops added) are not designable. Left: AF2 pLDDT is low (mostly below 80; the threshold for success; green area), and none of the AF2 predictions are within 2 Å of the cylinder (right, the threshold for in silico success, green area). *N* = 928 cylinders with unique n, S and I parameters, spanning the same parameter space as used in Figure 1c and d in the manuscript. For each backbone, 8 ProteinMPNN sequences were sampled, and the "best" sequence plotted (by RMSD between the AF2 prediction and design).

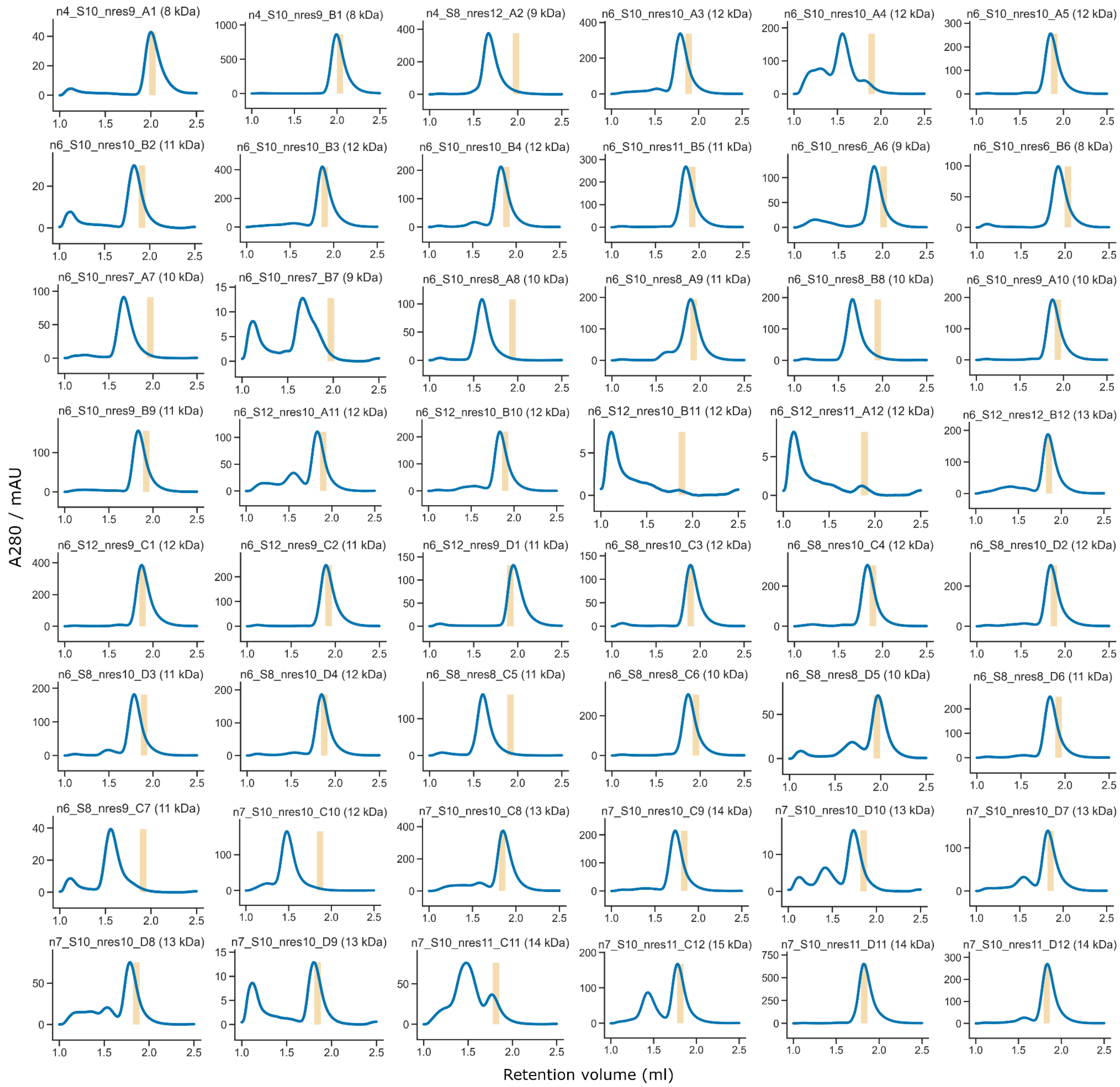
n4_S10_nres9_A1 (8 kDa)
n4_S10_nres9_B1 (8 kDa)
n4_S8_nres12_A2 (9 kDa)
n6_S10_nres10_A3 (12 kDa)
n6_S10_nres10_A4 (12 kDa)
n6_S10_nres10_A5 (12 kDa)
n6_S10_nres10_B2 (11 kDa)
n6_S10_nres10_B3 (12 kDa)
n6_S10_nres10_B4 (12 kDa)
n6_S10_nres11_B5 (11 kDa)
n6_S10_nres6_A6 (9 kDa)
n6_S10_nres6_B6 (8 kDa)
n6_S10_nres7_A7 (10 kDa)
n6_S10_nres7_B7 (9 kDa)
n6_S10_nres8_A8 (10 kDa)
n6_S10_nres8_A9 (11 kDa)
n6_S10_nres8_B8 (10 kDa)
n6_S10_nres9_A10 (10 kDa)
n6_S10_nres9_B9 (11 kDa)
n6_S12_nres10_A11 (12 kDa)
n6_S12_nres10_B10 (12 kDa)
n6_S12_nres10_B11 (12 kDa)
n6_S12_nres11_A12 (12 kDa)
n6_S12_nres12_B12 (13 kDa)
n6_S12_nres9_C1 (12 kDa)
n6_S12_nres9_C2 (11 kDa)
n6_S12_nres9_D1 (11 kDa)
n6_S8_nres10_C3 (12 kDa)
n6_S8_nres10_C4 (12 kDa)
n6_S8_nres10_D2 (12 kDa)
n6_S8_nres10_D3 (11 kDa)
n6_S8_nres10_D4 (12 kDa)
n6_S8_nres8_C5 (11 kDa)
n6_S8_nres8_C6 (10 kDa)
n6_S8_nres8_D5 (10 kDa)
n6_S8_nres8_D6 (11 kDa)
n6_S8_nres9_C7 (11 kDa)
n7_S10_nres10_C10 (12 kDa)
n7_S10_nres10_C8 (13 kDa)
n7_S10_nres10_C9 (14 kDa)
n7_S10_nres10_D10 (13 kDa)
n7_S10_nres10_D7 (13 kDa)
n7_S10_nres10_D8 (13 kDa)
n7_S10_nres10_D9 (13 kDa)
n7_S10_nres11_C11 (14 kDa)
n7_S10_nres11_C12 (15 kDa)
n7_S10_nres11_D11 (14 kDa)
n7_S10_nres11_D12 (14 kDa)
A280 / mAU
Retention volume (ml)

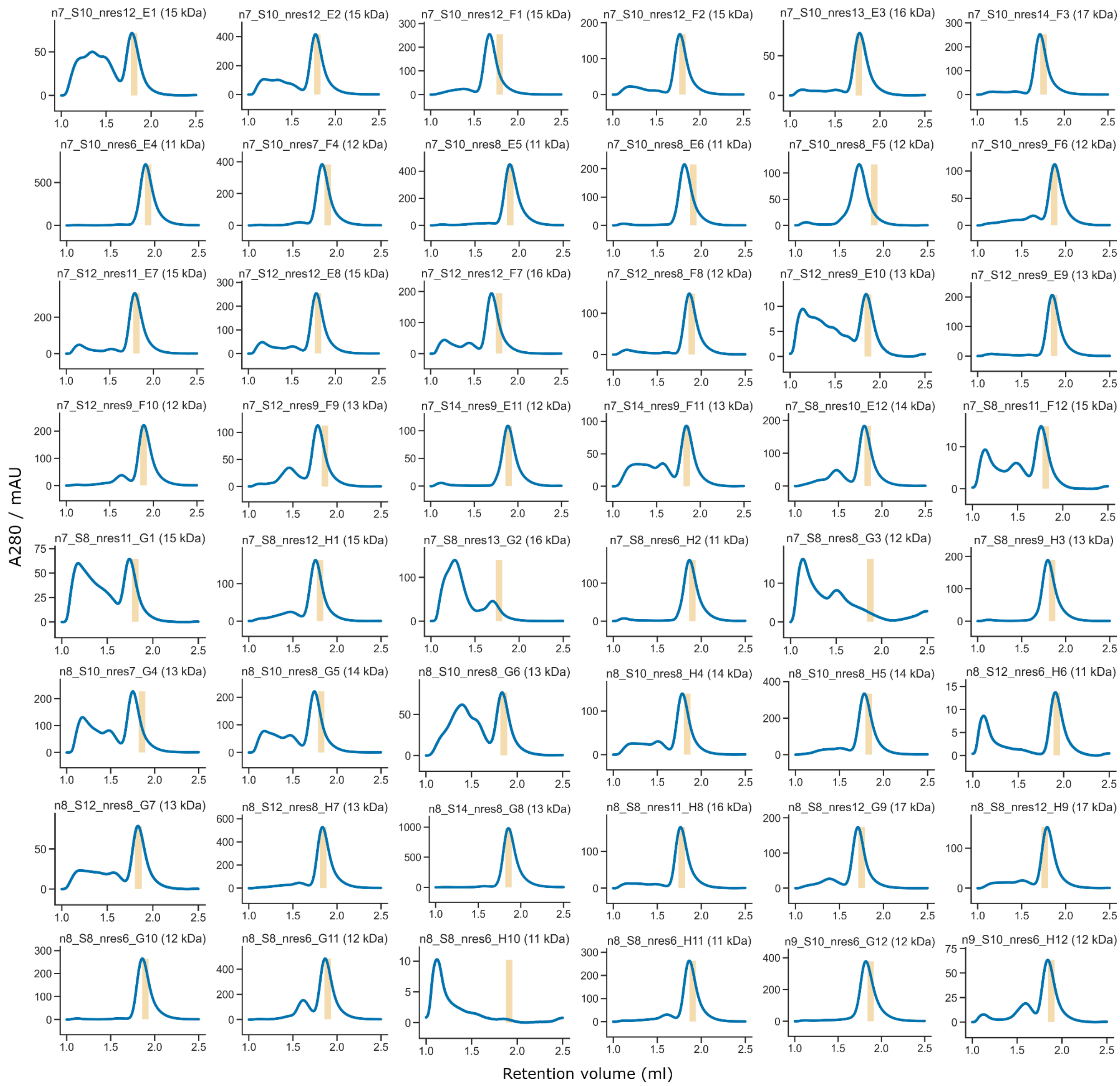

Fig. S4. Size exclusion chromatography traces with estimated retention volumes (vertical bars) for monomeric designs.

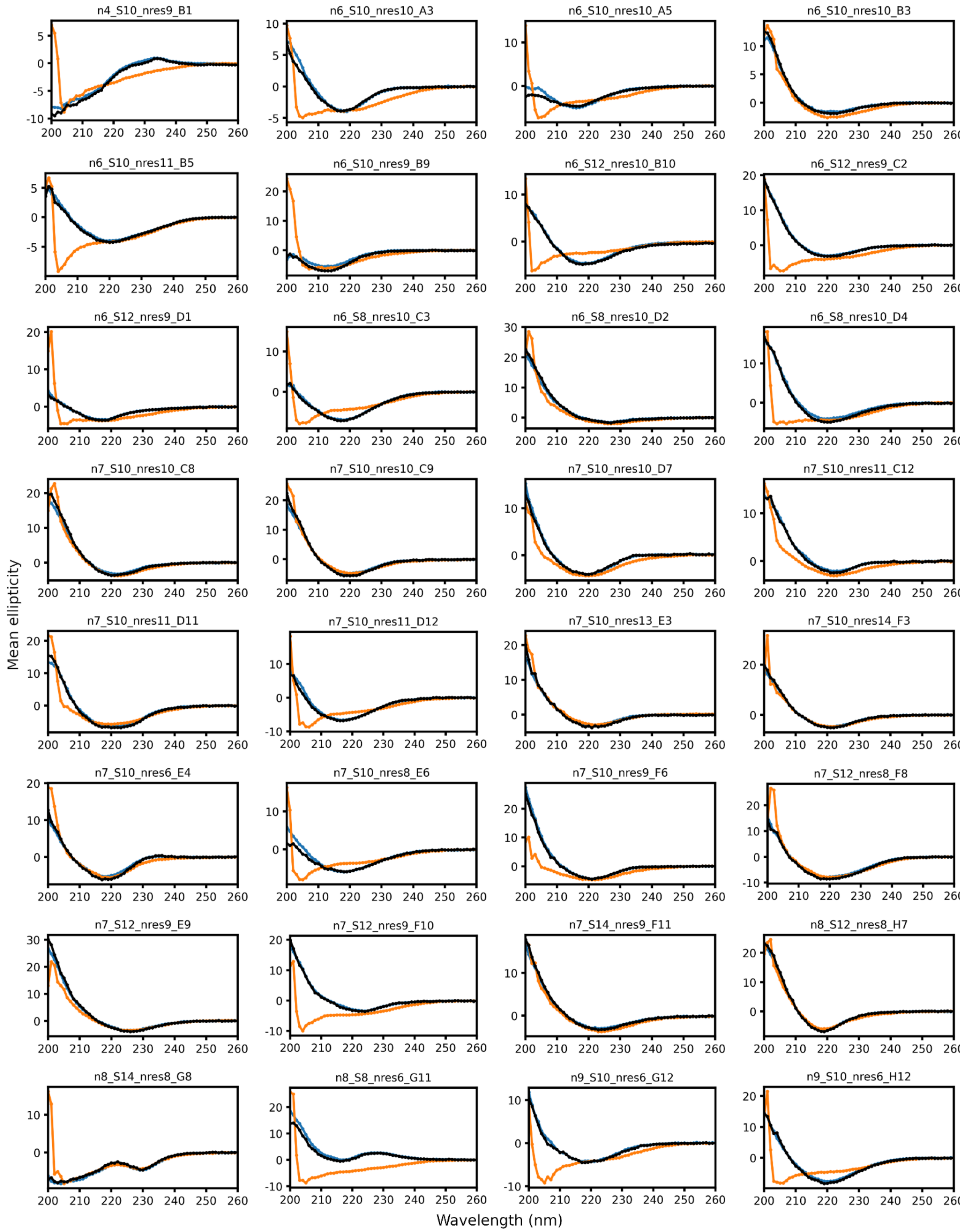

Fig. S5. Far-UV circular dichroism spectra of designs at 25 °C (blue), 95 °C (red), and back to 25 °C from 95 °C (black).

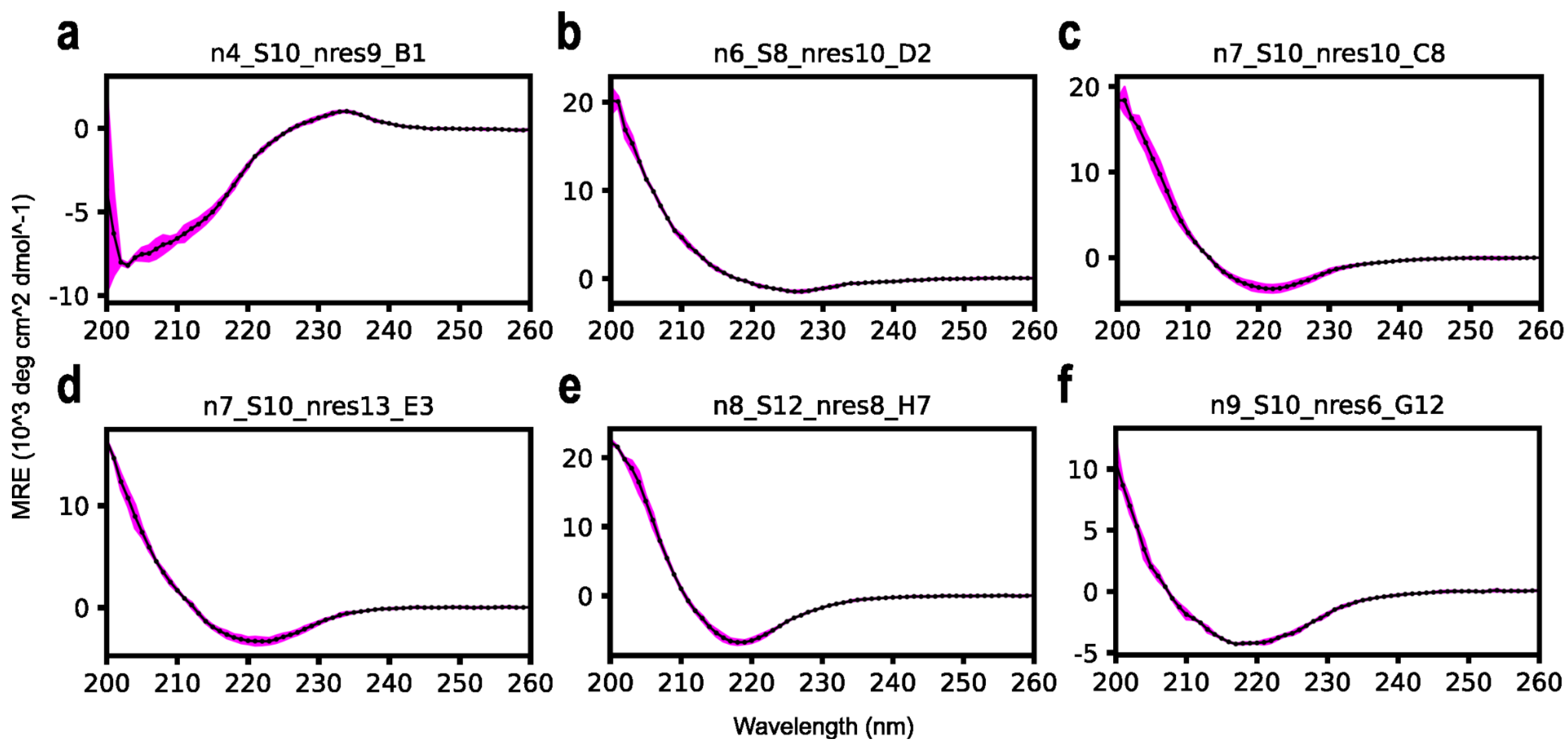

Fig. S6. Far-UV circular dichroism spectra of the designs in Figure 2 (**a-f**) from the main text at 25 °C (mean spectra in black with standard deviations shaded in magenta). Mean values were determined from two independent measurements each from separate expressed and purified batches. Protein concentrations were determined with predicted extinction coefficients using a NanoDrop spectrophotometer (ThermoScientific) to measure absorbance at 280 nm. Purified proteins were verified using liquid chromatography mass spectrometry (LC/MS).

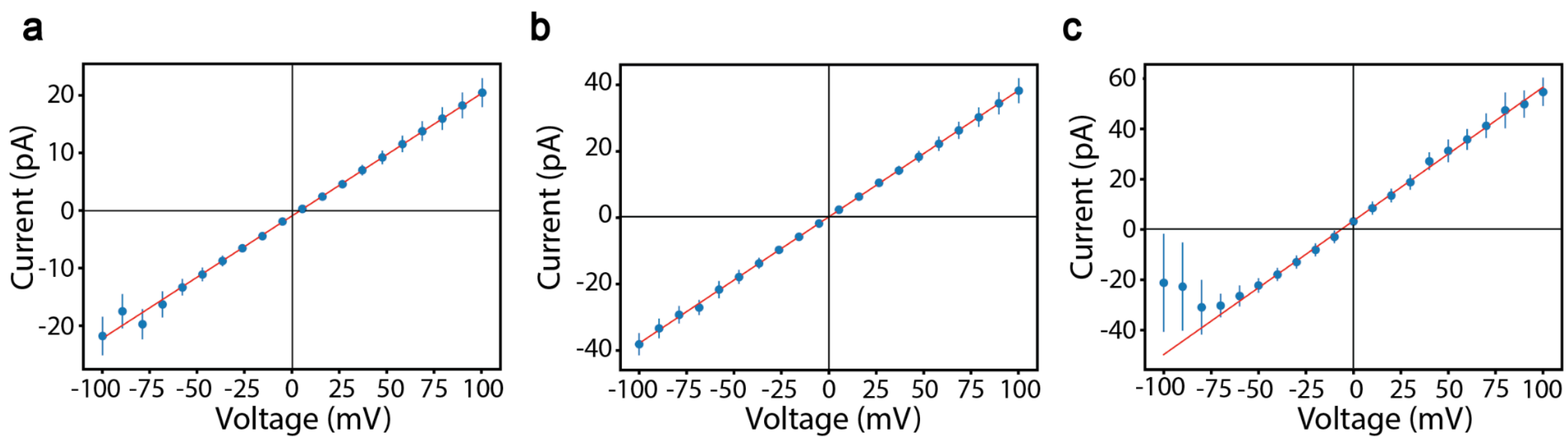

Fig. S7. Current vs Voltage curves for the lowest current jumps corresponding to single pore insertions of (**a**) TMB_n12_S14_nres14_1, (**b**) TMB_n14_S16_nres12_1 and (**c**) TMB_n16_S20_nres12_1. All measurements were carried out in a symmetric (same on cis and trans side of the bilayers) 500mM potassium chloride solution.

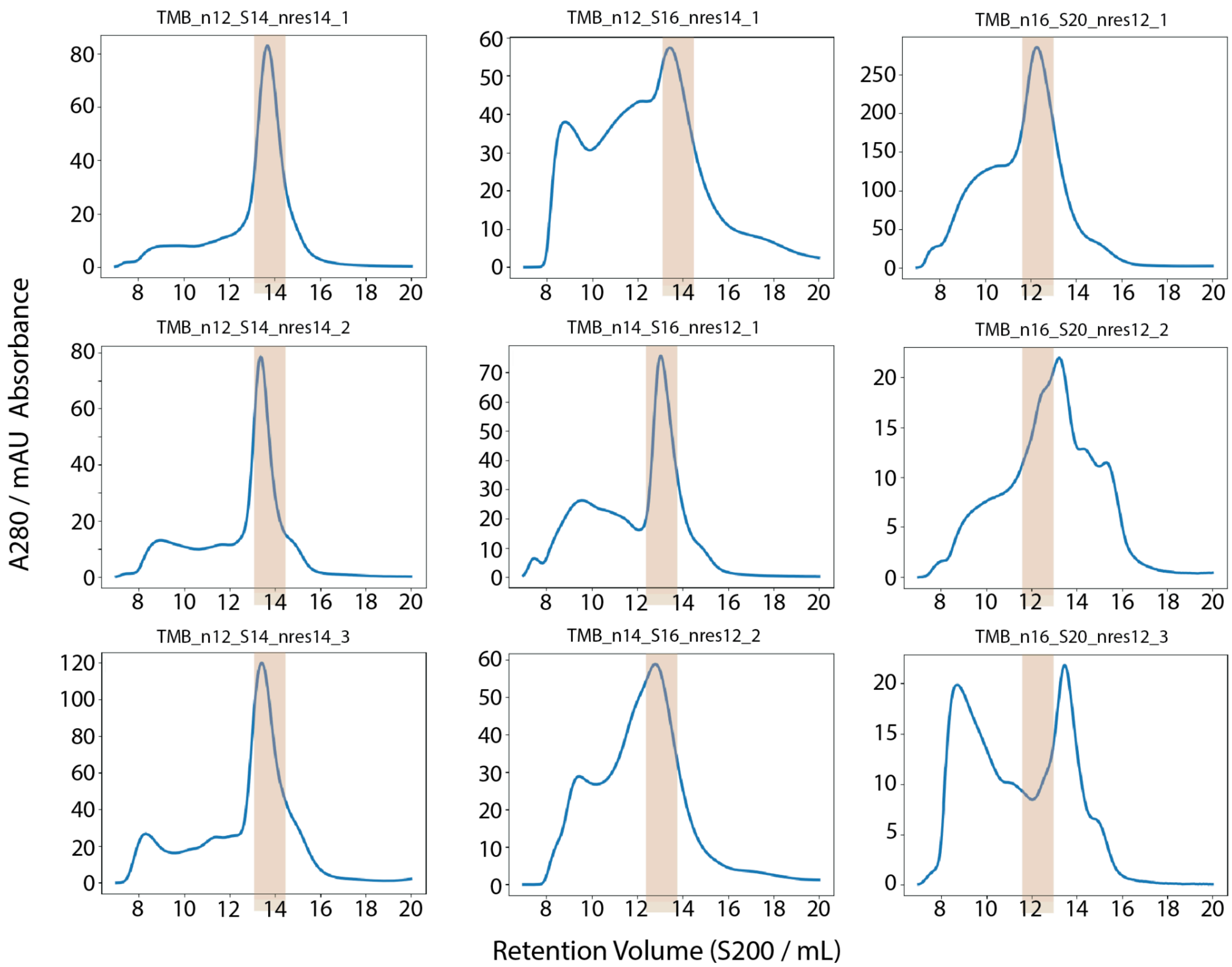

Fig. S8. Size exclusion chromatography traces with estimated retention volumes accounting for micelle size for DPC on a Cytiva S200 column.

Table S1. Benchmarking results for RFjoint2

Performance of RFjoint2 on the motif-scaffolding benchmark described in Watson et al. (3) The benchmark seeks to build de novo scaffolds around structural motifs. In RFjoint and RFjoint2, joint sequence-structure design is possible. In RFdiffusion (and RFjoint/RFjoint2 + ProteinMPNN (8)), 8 sequences of the scaffold region (but not the motif) are designed with ProteinMPNN. 100 samples are designed with each method, and the number of in silico successful (AF2 RMSD vs design model < 2 Å; AF2 motif RMSD vs native motif < 1 Å; AF2 pAE < 5) is plotted. Full benchmarking details are described in Watson et al. (3) Without ProteinMPNN, RFjoint2 "solves" 15/25 problems (compared to 6/25 in RFjoint), and outperforms RFjoint in 14 cases (with one tie). With ProteinMPNN, RFjoint2 "solves" 21 problems in total (compared to 23 in RFdiffusion and 17 in RFjoint) , and outperforms RFjoint in 15 of these cases. While RFdiffusion still generally outperforms RFjoint2, RFjoint2 outperforms RFdiffusion in 6 cases.

**Problem Name**	**RFdiffusion (noise=0)**	**RFdiffusion (noise=1)**	**RFjoint**	**RFjoint + Protein-MPNN**	**RFjoint2**	**RFjoint2 + Protein-MPNN**
1BCF	**100**	98	65	**100**	92	99
6E6R_med	**89**	67	0	27	10	38
2KL8	88	96	71	95	**100**	**100**
6E6R_long	**86**	63	0	4	24	52
6EXZ_long	**76**	51	0	0	2	8
1YCR	**74**	58	12	57	12	33
6VW1	**69**	66	0	24	35	63
5TPN	**61**	59	0	3	28	57
6EXZ_med	**49**	33	0	3	5	14
4ZYP	40	31	1	21	**53**	49
6E6R_short	**39**	29	0	23	7	19
5TRV_long	**37**	30	0	0	0	0
3IXT	35	16	21	62	77	**99**
5TRV_med	**24**	20	0	3	0	1
7MRX_85	**11**	6	0	0	0	8
7MRX_128	**9**	4	0	0	0	4
1PRW	8	9	0	22	19	**35**
5TRV_short	4	**7**	0	2	0	1
7MRX_60	**2**	0	0	0	0	1
6EXZ_short	2	4	1	**27**	2	4
5IUS	2	0	0	1	0	**12**
5YUI	0	0	0	**1**	0	0
5WN9	0	1	0	0	5	**46**
4JHW	0	0	0	0	0	0
1QJG	0	**2**	0	0	0	0

Table S2. DNA and amino acid sequences of experimentally characterized designs.

Design	DNA	AA
n4_S10_nres9_A1	CGTCTTAGACGCGACTCCCTTACTTGATTAGCCG CGTCGTTGAAAACatactacggtctcaaggaATGCGCGG CTATCGCGGCGATTTTGTGGATGAAGATGGCCGC GTTATTCCGGGTACCTATTATGGCGGCGAACGTC CGGTACCGGGTGAATGGGTGGAAGTTGTGGCGG AAGACGGCAAAACCATTCGCGCGCGTCTGGTGG GCGAACTGGAAGAATTTGAAGTTCCGggttcccgaga ccgtaatgcCGTTAGTTGACATAGTCGCGTGATATGA AACTTCTATGTCGGGATCA	MSGMRGYRGDFV DEDGRVIPGTYYG GERPVPGEWVEVV AEDGKTIRARLVGE LEEFEVPGSGSHH WGSTHHHHHH
n4_S10_nres9_B1	AGACGAGACGTCTTGCAAATTATCATCTTACGTAC AATCTAAAGTCAGCatactacggtctcaaggaGAAACCG GCTGGAGCGGCAAATGGGTGCTGGATGATGGCT CTGTGGTTGAAGCGACCTATTGGAACAACGGCGT GAAACCGAAGAAAGGCGAGAAATATGAAGTCGTG CTGCCGGATGGCACCGTGGCGACCGGTGAACCA GTTGGTGAACTGACCAAAGTGACCggttcccgagacc gtaatgcTGTCGACATTGACCGCTTTAATTTTCGCGC CGTACATACCTGAACCGG	MSGETGWSGKWV LDDGSVVEATYWN NGVKPKKGEKYEV VLPDGTVATGEPVG ELTKVTGSGSHHW GSTHHHHHH
n4_S8_nres12_A2	TACACCTGACGCTATGGACAACGTATatactacggtctc aaggaGCGAAATATACCGGCAAAGGTAAAGGCACC ATGACCAACGCGGATGGCAAAACCGTGGATGTG GAATTCACCGAATTTGAAGGTACCGGTACCCTGG AAGATGGCGTTCTGACCCTGACCAGCTGGAAAG GCAAAGGCGTTGCGGACGGCAAACCGTTCGAAG GCAGCGGCTCTGGTACCGTTACCGAAGGCGAAG TGAAAGCGGTGGAAggttcccgagaccgtaatgcCGGGG GTGAGGCTTGGGAGTGGCCTG	MSGAKYTGKGKGT MTNADGKTVDVEF TEFEGTGTLEDGVL TLTSWKGKGVADG KPFEGSGSGTVTE GEVKAVEGSGSHH WGSTHHHHHH
n6_S10_nres10_B2	atactacggtctcaaggaGCGCCGAAAGTGATGAGCGG CACTCTGACCGTTACTGGCGGTACTGGCATTAGC GGTGGCACCGTGGATGTGACCGGCGTGGTTGAT GATGGCGTGTTCACCGGTACGGGCACCTTTACG GGTACGGTTAACGGTAAAGCGGCGAGCGGTCCG GTGAACGTGGAAGCGGAAGTCGATGAAAACGGC GAAGTTACCGGTGGTACCCTGAGCGGTACCGTG ACGGGCGGTTATGTGATTAACGGCGCGGTTAAAC TGACCGGCACCCTGACCGAAGTGCCGGGCACCg gttcccgagaccgtaatgc	MSGAPKVMSGTLT VTGGTGISGGTVD VTGVVDDGVFTGT GTFTGTVNGKAAS GPVNVEAEVDENG EVTGGTLSGTVTG GYVINGAVKLTGTL TEVPGTGSGSHHW GSTHHHHHH

n6_S10_nres10_A3	atactacggtctcaaggaGCGAAACTGTATAAAGGCAAACTGAAAGTCACCGGCACCATCGGCGATACCAAAGTAGAAGGTGAAGTCGAAGCGTGGGGCGTGATTGATGGCGACACCACCGAATTTACCCTGAATGTTAAAGGCGAAGTGGAAGGTGGCACCATTGAAGGCGGCACCGTTAAAGCAACCGGTGAACTGACCCCGGAAGGCCTGAACGCGACCGGTACGTTTACGGGTACCTTTAACGGCGAAGAGAAAACGGGCGAAGTGACCCTGGAAGGTAAACTGGAAGAAGTTGAACCGggttcccgagaccgtaatgc	MSGAKLYKGKLKVTGTIGDTKVEGEVEAWGVIDGDTTEFTLNVKGEVEGGTIEGGTVKATGELTPEGLNATGTFTGTFNGEEKTGEVTLEGKLEEVEPGSGSHHWGSTHHHHHH
n6_S10_nres10_B3	atactacggtctcaaggaAAACTGAAAGTGGGTGAAGGCACCGGCGATGGCGAAGTGGGCGGCGTTAAAGTGAAAGATGTCAAAGTTCTGGTGGTGGGCAAAATTGAAGATGGCAAATTCGAAGGTGAAGCGACCATTACCGGCCTGCCGAACGGCAAAACCGCGAAAGGCAAAGCGGAAGGTAAAGTCGAAGGCAACAAAATTAAAGGCACGGTGACCGATATTGAACTGGATGGTAAGAAAGTTGGCGGTAAAATCGATTTTGAAGGCGAATACTGGGACATTGAAGAAggttcccgagaccgtaatgc	MSGKLKVGEGTGDGEVGGVKVKDVKVLVVGKIEDGKFEGEATITGLPNGKTAKGKAEGKVEGNKIKGTVTDIELDGKKVGGKIDFEGEYWDIEEGSGSHHWGSTHHHHHH
n6_S10_nres10_A4	atactacggtctcaaggaGAAAGCCTGAAATTTGAGGGCGAACTGGAACTGAAAGGTAACGTCGGTGGCAAAGAAGTTAAAGGTACGGTGAAGTTTGAAGGCGAACGTAAAGGCAACTACGTTGAGGGTGAATTCGAAGGCGAGCTGGAAGGCGGCATTAAAAGCGGCAAACTGGAAGGTGAAGGGGAGTTCGAGGGCGATTATTTCAAAGGCACCCTGACCGGCAACTTTGATGGTAAAGAAGGTACCGTCGAAGCGAAAGGCGAATTAAAATTCGAGGAACCGggttcccgagaccgtaatgc	MSGESLKFEGELELKGNVGGKEVKGTVKFEGERKGNYVEGEFEGELEGGIKSGKLEGEGEFEGDYFKGTLTGNFDGKEGTVEAKGELKFEEPGSGSHHWGSTHHHHHH
n6_S10_nres10_B4	atactacggtctcaaggaATGAAACTGTATAAAGCGCTGATTGAAGATCTGAAAGGTGAAGTCGGTGGCAAGAAAGTGGAAGGCGGCAAAGCGGAACTGATTGGCAACCTGGAAGATGGCAAATTTAAAGGTGAGGGCGAAGCCGAAGTGACCGTTGAAGGTAAGAAAGTCAAAGGCCTGGCGGTTGGCGAAGGCAAAGTCGAAGGCGAAAAGATTAAAGGCGAACTGAAATTTGAAAGCGATGACGGCGAGAAAGGCGTGCTGAAAGGGGAAGGCAAGGTCGAAGAAATTGAAggttcccgagaccgtaatgc	MSGMKLYKALIEDLKGEVGGKKVEGGKAELIGNLEDGKFKGEGEAEVTVEGKKVKGLAVGEGKVEGEKIKGELKFESDDGEKGVLKGEGKVEEIEGSGSHHWGSTHHHHHH
n6_S10_nres10_A5	atactacggtctcaaggaGAAGAAATTTATAAAGTTAAAATCACCGATCTCAAAGGTGATGTGGGCGGCAAAGTGGAAGGCGGTAACGCGGTGCTGGAAGGTGTGGTTAAAGATGGCATTTTCAGTGGTGAAGGTGAAGTCGAAGTGACCGTGAACGGCAAGAAGAAAACCGGTAAAGCGAAAGGCGAAGGCAAGGTGGATGGCGATGAATTTAGCGGCGAAATTGATGTTGAAGATGATGA	MSGEEIYKVKITDLKGDVGGKVEGGNAVLEGVVKDGIFSGEGEVEVTVNGKKKTGKAKGEGKVDGDEFSGEIDVEDDDGNKGKVKGKGKVFKL

	CGGCAACAAAGGGAAAGTGAAAGGCAAAGGTAA AGTGTTTAAACTGGATggttcccgagaccgtaatgc	DGSGSHHWGSTH HHHHH
n6_S10_nres11_B5	atactacggtctcaaggaGCACCAGTTGCGGTGAGCGG CACTGCGACCTTTACTAGCGGTACCCTGGGTGG CAAAGCGGTTGCAGGCGGCACCTTAACCGATATT ACGGGTACCGTGACCGGCACGACTTTTACGGGC ACCGCGACCTATACTGCCACCGATGGCACCACC GGTACCATTACCGGTTTAACCGCGGCCGTTGCGG ATGGTGCAATTACCGGCACAGCGACTGGTACCGA TACCGCAACCGGCCAGGCGGTTACGTTAACCGG TGTGAGTGGTACTCTGACCGCGGTTCCGGCGCC Gggttcccgagaccgtaatgc	MSGAPVAVSGTATF TSGTLGGKAVAGG TLTDITGTVTGTTFT GTATYTATDGTTGTI TGLTAAVADGAITGT ATGTDTATGQAVTL TGVSGTLTAVPAPG SGSHHWGSTHHH HHH
n6_S10_nres6_A6	TCGACGTCAAGTATAATCACCGCGAGGTCGGACC TatactacggtctcaaggaGCGCTGACCATCACCGGCAA AGTGACCATTAACGGCAAGGATGCGGGCACCGT GACCGGCGAAGGCGTTGAAGGCGAAGAATTTAC GGGCAAAGATGAAAACGGCGTGGAATTTAAAGG CGTGTATAAAGATGGCGTGCTGACCGGTACCCTG AATGGTGAGAAATTTAGCGGTCCGGCCACCGCG ACCGCGggttcccgagaccgtaatgcAGTCATAAGCGTTG GGGACTGTTTCGCGCCTTCAA	MSGALTITGKVTIN GKDAGTVTGEGVE GEEFTGKDENGVE FKGVYKDGVLTGTL NGEKFSGPATATAG SGSHHWGSTHHH HHH
n6_S10_nres6_B6	GCCCTACACCCCCTTACTTTGGAGTCGGGCAGA GatactacggtctcaaggaGCAACTGTGACCGGTACTGG TGGTACTCTGGGTGGTGCGCCGATTGCGGGCTT TAGCGGTACCTTGACCGATGGCGTGTTTACCGGC ACTGTGGATGGCAAACCGGCAACGGGTACTGTG GTGGACGGTGTTGCGAACTTTACCGTTGATGGTG TGGCGGGTACCGCGCCGGTGACTTCTATTACCCC AGCGCCGggttcccgagaccgtaatgcTGTAAGTCAATTC GTATCGACGCATAATCTACT	MSGATVTGTGGTL GGAPIAGFSGTLTD GVFTGTVDGKPAT GTVVDGVANFTVD GVAGTAPVTSITPA PGSGSHHWGSTH HHHHH
n6_S10_nres7_A7	CTTCACTGTCGAAGTATTTACAGTCatactacggtctca aggaGGCAAAGTGACCATTAAAGCGGAAGATGGC ACCGTTATTACCGGTGAAGCGAAAGCGATTGAAG AAAACGGCGTTACCCTGATCGAAGGCGAATTCGA AGGTGGTAAAGCGAGCGGCAAAGCCGAAAACCT GAAGAATGGCAAAACCGGCACGGGCACCATCAC CGTGAACGGTAAAGATTATACCGGCGAAATTTACT ATGAAGAAATTGAAggttcccgagaccgtaatgcGGGTTT AGTTCAACCCCCGTAGCA	MSGGKVTIKAEDG TVITGEAKAIEENG VTLIEGEFEGGKAS GKAENLKNGKTGT GTITVNGKDYTGEI YYEEIEGSGSHHW GSTHHHHHH
n6_S10_nres7_B7	CCATGAAAAGGCGGTAGCGAatactacggtctcaaggaG GCAAAGTGGTTGCAACCTATACCGGCCCGCCGA GTGGTGAAGGTACCGTGACCGGTGAAATTGTGG GCGATGGCTTTGTTGGCACTGATGCGGCAACCG GTGCAACTGTGCGCGGCCCGTTAGTTCCAGTTG GCGAAACCGGCGAAGGCGAATACGAAGATGATG	MSGGKVVATYTGP PSGEGTVTGEIVGD GFVGTDAATGATV RGPLVPVGETGEG EYEDDGVWVSGFT YTPTEIEPAVAGSG

	GCGTTTGGGTGAGCGGCTTTACGTATACCCCGACCGAAATTGAACCGGCCGTGGCGggttcccgagaccgtaatgcTTGCACTAAGTGCCGGGGGC	SHHWGSTHHHHHH
n6_S10_nres8_A8	CTTGAGTTTGTCTCatactacggtctcaaggaATGCGCGTGTTAGAAGTGGATAGAGGCGAAGTGGGCGGCGAAGAAGTTGAAGGCGGCCGCGTAGAAGGTGATGTCGAAGAGGGTGAAACTGGTCCAGCGCGCGGTGAAGTTGGCGGTGTTCCAGCCGAAGGCGAATTATATGTTGAAGATGGCCGAGGTCGTGTGGAAGGCGAGGCGGATGTTGATGGTGAACGTGTGCCGTTTCGTGCGGAAGGCCGTGTCGTTGAACCGCCGggttcccgagaccgtaatgcGTGGCATCTTATCG	MSGMRVLEVDRGEVGGEEVEGGRVEGDVEEGETGPARGEVGGVPAEGELYVEDGRGRVEGEADVDGERVPFRAEGRVVEPPGSGSHHWGSTHHHHHH
n6_S10_nres8_B8	AGGCCATGGGATTGCCAGAatactacggtctcaaggaACCGTGTATGAATTTGAAGTTGAAGGTGGCACCAACGGCCTGACCGGTGGCAAATTTGAGGGCGAACTGGAAGGCGATAAAGTTAAACTGAAAGGTAAAGACGATAACGGCGTTGAAGTCGAAGGTGAAGGCACCCTGAAAGATGGCAAAGCGAAAGTGGAAGGCAAAGACGAAAACGGCAAACCGGTTGAATTTACCGCGAAATTTGTGAAAGAGCTGGAAAAGggttcccgagaccgtaatgcAAGGCTGTTCTATATTCA	MSGTVYEFEVEGGTNGLTGGKFEGELEGDKVKLKGKDDNGVEVEGEGTLKDGKAKVEGKDENGKPVEFTAKFVKELEKGSGSHHWGSTHHHHHH
n6_S10_nres8_A9	AACAGCTCatactacggtctcaaggaGGCATGGATCTGGTGGAAGGTGAAGTCGAAGGCACCGTGGCGGGCAAACCGGCAAAAGGCGAATTTAAAGGTGAACTGAAAGACGGCAAAGTCAAGGGCGAAGTTAAAGTTGAAGTGGATGGCAAAGAAGTGAAAGGCCTGGCGGAAGGTAAACTGTATGAAGGCAACTACGTTAAATTAGAAGGCAAAACCGATGATGGCACCGAAGGCCTGAAAGCCGAGGGCAAAGTTAAGGTGATTAAACCGggttcccgagaccgtaatgcTAACCGAC	MSGGMDLVEGEVEGTVAGKPAKGEFKGELKDGKVKGEVKVEVDGKEVKGLAEGKLYEGNYVKLEGKTDDGTEGLKAEGKVKVIKPGSGSHHWGSTHHHHHH
n6_S10_nres9_B9	GGCTTCGatactacggtctcaaggaGCGACCAAAGTGACCATTGAAGGTACCCTGGAAACCGATGACGGCTATGTGGGCGAAGTGAGCGGCGAAGGCGTGTTATTAGAAGGCGGCAAAGGCGTTTTCGATCTGACCGATACCGAAACCGGCGAGAAATTTACCGTCGAACTGAAGAACGCGAAACTGGGCGAGGGCACCTTCGAAGTTACCAAAATTATTCGCGATGGCAAAGATATTGGCGGCGGCACGTTTACCGGCACCATTACCGAAGTGggttcccgagaccgtaatgcGTAAGT	MSGATKVTIEGTLETDDGYVGEVSGEGVLLEGGKGVFDLTDTETGEKFTVELKNAKLGEGTFEVTKIIRDGKDIGGGTFTGTITEVGSGSHHWGSTHHHHHH
n6_S10_nres9_A10	AACGCACACTatactacggtctcaaggaAAATATCTGCTGGAAGGCGAATTTACCGATGCCGATGGTACCAAAGGCACCATTAAAGCGGAAGGTGAAGTTAAAGATGGCGTGTTTACCGGCGACGGCACCGCGACCGTGGATGGCAAGAAATATAAAGTCACCGGCGTCAAAGGCGAAGTGGACGGCGATAAATTTAAAGGCAAAACCG	MSGKYLLEGEFTDADGTKGTIKAEGEVKDGVFTGDGTATVDGKKYKVTGVKGEVDGDKFKGKTGDFSDGTAGGAPVEGT

	GTGATTTTAGCGATGGCACTGCGGGCGGTGCGC CGGTTGAAGGTACCGTGAAGAAAGTGGAAggttccc gagaccgtaatgcCCTTGAGAT	VKKVEGSGSHHW GSTHHHHHH
n6_S12_nres10_B10	atactacggtctcaaggaGTGGAGAAATTTAAAGTCGTG GGCAAAGTGATTAGCGGTACCGTGGGTGGCGAA GCGGTGGAAGGTGGTACCATTGAAGGCGAAGCC ACCAAAGAAGGCGACGAAACCACCATTACCGGC AGCTTTACGGGCACCGTTGGCGGTGAAAACGTG AGCGGCAGCAACCTGACGTTTAAAGGCAAACTG GATGGCAACAAATTTACCGGTACCCTGACCGGCA CCATTGGCGGCAAAACCTATACCGGCGGCGAACT GACCTTTGAAGGTGAACTGATTGAACCGggttcccga gaccgtaatgc	MSGVEKFKVVGKVI SGTVGGEAVEGGTI EGEATKEGDETTIT GSFTGTVGGENVS GSNLTFKGKLDGN KFTGTLTGTIGGKT YTGGELTFEGELIE PGSGSHHWGSTH HHHHH
n6_S12_nres10_A11	atactacggtctcaaggaATGAAAACCTTCAGCGGCACC TTAACCTTTACCGGCACCCTGGGCGGTCAGGCG TTAACCGGTGCAACCGCAGAAGTTTCTGGCGTG CTGGAAGGCGATACCCTGACCGGTGAAGGCACT CTGACCGGTACCATTGGCGGCAAAGCGGTTAGC GGCCCGTTTACCTTTACGGGCAAATATACCCCGG GCCAGGATTTTCAGGGCACGCTGACCGTGCAGG TGGGCGATAAAACCGTGAGCGGCGATGTGACCT TTAGCGGTGAACTGGAATTTACCGATCTGggttcccg agaccgtaatgc	MSGMKTFSGTLTFT GTLGGQALTGATAE VSGVLEGDTLTGE GTLTGTIGGKAVSG PFTFTGKYTPGQD FQGTLTVQVGDKT VSGDVTFSGELEFT DLGSGSHHWGSTH HHHHH
n6_S12_nres10_B11	atactacggtctcaaggaCGCACCCGCGTGGTGGCGCG CGTGCGTCTGACCATCGATGGTAAAGAACATCGC GGCGAACTGACCTTTGATGGTGAAGTGACCGAA GGCGAAGGCTTTCGCGGCACCTTTAGCGGCACC ATTGATGGCAAACCGGCGAGTGGTGAACTGGAA GGCCGTGGCGAAATTACCGAAGAACGCTTCCGC GGTGAAGCGACCGGCACGATTAACGGCCAGAAA ATTGACGGCGCGCCGATTGATGCGGAAATTGTGG AACGCGAAGAAATTCCGCCGggttcccgagaccgtaatg c	MSGRTRVVARVRL TIDGKEHRGELTFD GEVTEGEGFRGTF SGTIDGKPASGELE GRGEITEERFRGEA TGTINGQKIDGAPID AEIVEREEIPPGSG SHHWGSTHHHHH H
n6_S12_nres11_A12	atactacggtctcaaggaGCGCGCCGTGTTCGCGCGAC TGGCACCGTTGAAGTTGATTTGCCAGGTGGCCG TCGTGCGACCATCGAATTTGATGGTGAAGGCGAA CTGGAAGGTGATACCCTGGTGGTGCCGTTCACC AGCGGCACCATTGATGGCAAACCGTTTACCGGC GAAGGCACCTTAACCATTCGCGGTGATCCTGAAG CGCCGGGTGCACCGGTTACTGCAACTTTCACCG GTGATGCGGGTACCTTTGTGGATTATCCAGGTCG CGTGGTTGCGGCGCGTGTGACTCCGTTACCGGC Gggttcccgagaccgtaatgc	MSGARRVRATGTV EVDLPGGRRATIEF DGEGELEGDTLVV PFTSGTIDGKPFTG EGTLTIRGDPEAPG APVTATFTGDAGTF VDYPGRVVAARVT PLPAGSGSHHWGS THHHHHH
n6_S12_nres12_B12	atactacggtctcaaggaATGAAAGGCTTTGTCGCCGAA GGCGAAATCACCAACGGCAGCTTTAACGGCAAA	MSGMKGFVAEGEI TNGSFNGKPVKGK

	CCGGTGAAAGGCAAATTTAAAATGAAAGTGATTGGCGAACTGGGCAAAGGGAAAAGCGCGCCGGCGGAATTTGATATTATTAACGAAAACGGCGAAGTTGTGGGCCGCTTTAAAGGCACCATTTATTTTGGCGATATTGATGGCAACAAATTCACCGGCAAATTCGATGCGGAAGGTACCCTGGGCGATGAGAAAGGTAAATTTAGCGGCGAATTCGAAGCGGAAATTACCGAAACCTTTGAACTGggttcccgagaccgtaatgc	FKMKVIGELGKGKSAPAEFDIINENGEVVGRFKGTIYFGDIDGNKFTGKFDAEGTLGDEKGKFSGEFEAEITETFELGSGSHHWGSTHHHHHH
n6_S12_nres9_C1	atactacggtctcaaggaGGCAACTATGTGAAATTCAAAGTGTATGATGAAAACGGCAACCTGCTGGGCGAAATTTACATCGAAACCGATGAGAACTTCGGCAAGAAAGGCTATAAAAGCAAAATTAGCGGCGTGATTAATGGCAAACCGATTGATGAAGCGGAAGTGACCTTTCTGACCGATATCGAAAATGGTAAAGCGAAAGTGATTATCAGCGGTGTGATTGGCGGCGAGAAAATTGAAAACAAAGTGGTCAACATCGAATTTATCGAGAACGTGGACGAACCGggttcccgagaccgtaatgc	MSGGNYVKFKVYDENGNLLGEIYIETDENFGKKGYKSKISGVINGKPIDEAEVTFLTDIENGKAKVIISGVIGGEKIENKVVNIEFIENVDEPGSGSHHWGSTHHHHHH
n6_S12_nres9_D1	TTTAGatactacggtctcaaggaATGACTGTGGAAGTGGCGACTGTTACCGGCGAAGGTGGTGCGGTTGCACCGGGTTCTACTTTGACCGGCACTCTGGAACCGGGTCAAACCGTGGATTTTAGCGGCGTGTTTCGTGGCCGTCCGGCGACCGGTCGTTTAGAACTGCTGACTCGCCCTGAAGTTGGCAAATGGTTTGATGCGCGTGGCACCATTGGCGGCGTTGAAGTTCCGCGTCTGCGTCTGTTCGTGGAAGAAATTCGCACCGTCGAAGTGggttcccgagaccgtaatgcGGTTC	MSGMTVEVATVTGEGGAVAPGSTLTGTLEPGQTVDFSGVFRGRPATGRLELLTRPEVGKWFDARGTIGGVEVPRLRLFVEEIRTVEVGSGSHHWGSTHHHHHH
n6_S12_nres9_C2	GatactacggtctcaaggaAAGAAATATAAAGTTACCGCGAGCGGCACGGCGAACGGCCAGGCGTTTGATGAAGTTGTGTTTACCGTGCCGAGCCTGAAGAAAGGTGCAGTAGCGCCGGTTACCGGTACCGTTGGCGGTGTGGCAATTGCGTCTGGTACCGTGACCTTTCTGGATGATGTGGAAGTGGGCAAAACGTTTACCTTTACCGGCACCCTGAACGGCGTTGCGATTACGGGTGGCACCGCAACCGTGAAAAGCATCGAAGAAATTGAAGCGGAAggttcccgagaccgtaatgc	MSGKKYKVTASGTANGQAFDEVVFTVPSLKKGAVAPVTGTVGGVAIASGTVTFLDDVEVGKTFTFTGTLNGVAITGGTATVKSIEEIEAEGSGSHHWGSTHHHHHH
n6_S8_nres10_D2<br>(BBn6 in main text)	atactacggtctcaaggaAAGAAAGTGAAATTCGTGGCAGAAGGCACCTTTAAAGCGACCAACAAACCGATTGAAGGCCTGGTTAAAGCCGAAGGCGAGGCGGAACTGGGCAAAGATTTTGAAGCAGAAGTGACCGGCGAACTGGATGGCAAACCGTTTAAAGGCAAAGCGAAATTTCCGCCGTACAAAGGTAAAGGCTATACCTATCGCGGCGAAGCGGAAGGCGAAATCGATGGCGAACCGATTAAAGGTATTGTGGATGCGACCGTGTTCAAAGAAGAGGAAGTTCCGGAAGGTGAAggttcccgagaccgtaatgc	MSGKKVKFVAEGTFKATNKPIEGLVKAEGEAELGKDFEAEVTGELDGKPFKGKAKFPPYKGKGYTYRGEAEGEIDGEPIKGIVDATVFKEEEVPEGEGSGSHHWGSTHHHHHH

n6_S8_nres10_C3	atactacggtctcaaggaCGCGTGCTGGTTAGCGGTGAAGGGGAATTCGAAGGCACCCTGGGCGGCAAACCGGCCAAAGGTACCGTTCGTGTGGAAGATGCCGAGGTTGAAGGTGATCGCGCACGTGGCCCGGCAACAGTTACCGCAGATGGCAAAACCGGCGAAGTGGAAGCGGATCTGGAATTACGCGGCGATGAACTGCGCGGCCGTGTTCGTGGCGAAATTGAGGGTGAACCGGTAGAAGGCGAAGCGCGTGTGCGTGTTACCGAACGTCGCGAACTGCCGCCGggttcccgagaccgtaatgc	MSGRVLVSGEGEFEGTLGGKPAKGTVRVEDAEVEGDRARGPATVTADGKTGEVEADLELRGDELRGRVRGEIEGEPVEGEARVRVTERRELPPGSGSHHWGSTHHHHHH
n6_S8_nres10_D3	atactacggtctcaaggaAAAGAAATTATCGTTAACGGCAAAGGCACCGCCGAAGGCAAACTGGGTGGCAAAGAAGTGACCGGAGAAGCGGTGCTGGAAAACGGCCGCCTGGAAGGCGATAAAATTACCGGCAAAATTCTGGTGGAAATGGATGGCCAGAAAGGCGAAGTCGAAGCGGAACTGACCCTGGAAGGTGATAAAGCGACCGGCCCGGTTAAAGGTGAAGTGGGCGGCAAACCGTTTGAAGGCGAGGCGACCATTGAAGATCTGGAACTGGAAGAAGTGGAAggttcccgagaccgtaatgc	MSGKEIIVNGKGTAEGKLGGKEVTGEAVLENGRLEGDKITGKILVEMDGQKGEVEAELTLEGDKATGPVKGEVGGKPFEGEATIEDLELEEVEGSGSHHWGSTHHHHHH
n6_S8_nres10_C4	atactacggtctcaaggaGAAGAAAAGCTGTTTAAATTTGAAGGTACCTTTACCGGCGACGGCGGCAAACTGAAAGGTAAAGTTAAAGGCGAAGGCTATGCGAAAGAAGGCGATACCAGCTTCGAAGGTGAAGCGAAAATTTATCCGGATGATGGCGGCCCGGTGATTAGCGGCAAAGCCAAAGTTGAGGGCGAAATTAAAGATGGCTTTAAAGGTGGCACCGGCACCTTTGAAAACGAAGACGGCGTGAAAGGCACCATTACCGATTTAAAAGGCAACCTGGAAGATATTGAAggttcccgagaccgtaatgc	MSGEEKLFKFEGTFTGDGGKLKGKVKGEGYAKEGDTSFEGEAKIYPDDGGPVISGKAKVEGEIKDGFKGGTGTFENEDGVKGTITDLKGNLEDIEGSGSHHWGSTHHHHHH
n6_S8_nres10_D4	atactacggtctcaaggaGGCCGCCTGCGTGGCGAGGGCGAATTCGAAGGTGAAATCGGCGGCGAAAAGGCGAGCGGTCCGGTTACCGTTGAAGGTGAAGCGGAACTGCGCGATGGTGAATTACGCGCGCGTGTTGAAATTAAAGGCGAAGCGAACGGCGGTGAGAAAATTGAAGGAGAAGGCGTTTTACGCGGTCGCCTGGAAGGTGATACCGTTCGCGGTGAACTGGAAGGCGAACTGAACGGCAAACCGTTTCGCGGCGAAGTGGTGTTACGCGATGCGCGCTTTGAAGAACTGCCGGAAggttcccgagaccgtaatgc	MSGGRLRGEGEFEGEIGGEKASGPVTVEGEAELRDGELRARVEIKGEANGGEKIEGEGVLRGRLEGDTVRGELEGELNGKPFRGEVVLRDARFEELPEGSGSHHWGSTHHHHHH
n6_S8_nres8_C5	GGGTGTGatactacggtctcaaggaGAGAAACGCGTTTTCCGTGGCGAGGGTGAAGGCGAACTGGAAGGAGAACGCGTCGGCTTTCGTGCAGAATTAGCGCTGCCAGGTGTTGGTCAGGTGGGCCGCGGCGAAGTTGAAGATGAAGAAGGCCGTCGCGGTGAAGCAGAAGGCGTGCTGTTAGATGAGGATGGCAGCCGTATTCTGGTGCGTGGTGAAGTGGGCGGTGTGCCGTTTGAAGGTGAACTGTTTGGCACCGAAGATATTGGTAC	MSGEKRVFRGEGEGELEGERVGFRAELALPGVGQVGRGEVEDEEGRRGEAEGVLLDEDGSRILVRGEVGGVPFEGELFGTEDIGTVGSGSHHWGSTHHHHHH

	CGTGggttcccgagaccgtaatgcACATAC	
n6_S8_nres8_D5	CCGGACGTTGTATACGatactacggtctcaaggaATTGTT GGCACCGGCACCCTGACCACCAACCTGGGCACC CATACCGGCAATATTACCGTGACCGGTACCGATAA GAACAACATGACCATTAGCGGTACCCTGGATGGC CAGGATTTCTCTGGCACGGGCACCGTGAACGAT GATCTGAGCAGCGGCACCTATAGCGGCACGCTG AACGGCCAGTCTGTTAGTGGCACCTTTAAACTGA CCAACATTAACAAAATTCCGGGCggttcccgagaccgta atgcGTGCGTTCAGAAGAC	MSGIVGTGTLTTNL GTHTGNITVTGTDK NNMTISGTLDGQD FSGTGTVNDDLSS GTYSGTLNGQSVS GTFKLTNINKIPGGS GSHHWGSTHHHH HH
n6_S8_nres8_C6	CTAGACACTTTAGCTGCatactacggtctcaaggaATGGC GGTGTTTAAAGGCACCGTGGAACTGGATGGTGG TGAAGTGGGCACCTTTGAAGCGGAAGGCAAACC GGGCGAAACCGTGCCGGTCAAAGGCGAAGTGG ATGGCGAACCGATTGAAGGCGAGGGCGTTATTAG CGAAGATGGCAAAAGCGGTACCTTCAAAGGTGAA TATCGCGGTAAACCGGTGACCGGCAAACTGAAA GTGACCGAAGTTGAAGTGCCGCCGggttcccgagacc gtaatgcTTTTAAGTTGTGAAAAA	MSGMAVFKGTVEL DGGEVGTFEAEGK PGETVPVKGEVDG EPIEGEGVISEDGK SGTFKGEYRGKPV TGKLKVTEVEVPPG SGSHHWGSTHHH HHH
n6_S8_nres8_D6	CGCAATCGatactacggtctcaaggaGAAACCCTGCTGC AGCTGACCGGTGAAGGCGATTTCGATGGCGTGA AATTTACCTTTGTGACCGAACCGTTTGCGCCGGA TGCGACCACCGTGAACTTTACGGGCACCACGGA AGATGGCACCGTCTTTACCGGCACCGCGACCTTA AATCCGGATCGTACCGGTGGCACTGGTGAAGCG ACCATTAACGGCGAGAAAGTGACCGGCGAATTTA AAGTTACCGAAGTGCGCGAAGTTCCGGCGCCGg gttcccgagaccgtaatgcTTTCGCTG	MSGETLLQLTGEG DFDGVKFTFVTEPF APDATTVNFTGTTE DGTVFTGTATLNPD RTGGTGEATINGEK VTGEFKVTEVREVP APGSGSHHWGST HHHHHH
n6_S8_nres9_C7	atactacggtctcaaggaGCGGTGGTGGAAGATTTTAAA GCGGAAGGCGAACTGGACGGCAAACCGGTGAC CGCGGATTTTGGCACGGTGGATTATGAAGAAGGC AAAGGCGTTAGCGGTGAAGGCACCGTGACCATT GATGGCAAGAAAGTGCCGGGCAGCTTTGATGGT GATTTTGATGCGGAAACCCAGAAAGCCAAAGGTA CCATGAAAGCGACCCTGGATGGTAAAGAACTGAG CGGCGAAGCCACCGGCAGCGGCAAAGTTGTGG AACTGGAAGGCggttcccgagaccgtaatgc	MSGAVVEDFKAEG ELDGKPVTADFGTV DYEEGKGVSGEGT VTIDGKKVPGSFDG DFDAETQKAKGTM KATLDGKELSGEAT GSGKVVELEGGSG SHHWGSTHHHHH H
n7_S10_nres10_D7	atactacggtctcaaggaGCGACCGCGACCCTTACCCTG ACCGTGACCGATGAAAACGGCGAAACCCTGACG CTGCAGCTGGATCTGACGGATTTTGATGCGGATG GTGTGGCAACCCCGACCTTAACCGCAGTGAGTG GTGGTCGTACCCCAGGTAGCGCAAGCGGCAAAG TGACCATTAAAGATGGCAAACTGACCTTTGATCTG ACCCTGAGCTATGAACGCGATGGCGTGCGTTATG AAGGCCGCCTGAAAGGCGAAGGCACCTACGATG	MSGATATLTLTVTD ENGETLTLQLDLTD FDADGVATPTLTAV SGGRTPGSASGKV TIKDGKLTFDLTLSY ERDGVRYEGRLKG EGTYDAATGTATAT ATEGVLRRVEAGS

	CAGCAACCGGTACTGCGACTGCAACCGCCACCGAAGGTGTTCTGCGTCGCGTGGAAGCGggttcccgagaccgtaatgc	GSHHWGSTHHHHHH
n7_S10_nres10_C8	atactacggtctcaaggaGCGATTGATGCGGAAGGCACCTTTAAAGGTACGGTCGATGGTAAAGAAGTTACCGACGGCAAAGTGAAAGTGACCGGCCTGGTGCTCGAAACCGGCTATGAAGGCAAAGCGAAAGTTAAAATTCAGGTGGATGGCAAATGGCTGGGCGAAGGTACCCTGAGCTATAAAGTCACCGAATACAAAGACGGTAAGTTCAAATTCGACTTCACCATCACCAACCTGGATAACGGCAAATATAAAGGCAGCGGCACCGCGACCGGCGAAGTGGAAGGCGATAAAGCCAAAATTGATGTGAAAGGCACCATCGAAAGCACCGAAggttcccgagaccgtaatgc	MSGAIDAEGTFKGTVDGKEVTDGKVKVTGLVLETGYEGKAKVKIQVDGKWLGEGTLSYKVTEYKDGKFKFDFTITNLDNGKYKGSGTATGEVEGDKAKIDVKGTIESTEGSGSHHWGSTHHHHHH
n7_S10_nres10_D8	atactacggtctcaaggaGCGAAGAAATTTACCGGCACCTTTAAAGCGACCGCGACGATTGATGGTAAGAAAGTGACCGGCGAAGGTGAAGTGGAAGGCGAAGTTAAAGATGGCGAAGTCACCTTCAAAATTACTAGCGGCGAACTGGGCGGTAAACCGATTGAAGGCGGTGGCGGCAAAGGCAAAGTGGATGAAAACGGCAACATTACCATTGATGAAGTTACGGCGACCATTGGTGGCGTTCCGGCGGAGAAAGGTACCGGGGAAGGTAAACTGTTTGATGATGGCACCGTGGAACTGAAACTGAAATTAGGCGCGGTGGATACCAGCGGCATTAAAggttcccgagaccgtaatgc	MSGAKKFTGTFKATATIDGKKVTGEGEVEGEVKDGEVTFKITSGELGGKPIEGGGGKGKVDENGNITIDEVTATIGGVPAEKGTGEGKLFDDGTVELKLKLGAVDTSGIKGSGSHHWGSTHHHHHH
n7_S10_nres10_C9	atactacggtctcaaggaATGCGCGTGGAAGGCGAACTGGTTGTGATTGATGAAGATGGCACCCGCTATGAAGGCCCGGCGGTTCTGGATCTGGATGAGGATGGCAAAGGTACCCTGAAAGCGAAACTGTATCGCGATGGTAAACTGGTGCTGGAATTCGAAGGCGAGATCGAACTGGGCGAAGACGGCAAAACCTTTACCGTTGAAGCCGAAGGCACCCTGGAAGGTAAAGAAGTTCGCTTTCGCGGCGAAGGTGAAATCGAAATTGAAGGCGATAAATTTAAAGCGGAACTGCGCGCGGAATTTGAAGAAGTGGAACCGCCGCCGggttcccgagaccgtaatgc	MSGMRVEGELVVIDEDGTRYEGPAVLDLDEDGKGTLKAKLYRDGKLVLEFEGEIELGEDGKTFTVEAEGTLEGKEVRFRGEGEIEIEGDKFKAELRAEFEEVEPPPGSGSHHWGSTHHHHHH
n7_S10_nres10_D9	atactacggtctcaaggaGAACCGTTTCGCTTTACCACCAGTGTGGATGGTACGCTGGATGGCACCGCCTTTACCAGCGGCACCGTGGATCTGAGCGTGTCTGATGTGGGCGATGGCAAAGCCACCATCACCTTCGATAGTGGCACCTTTGCGGGTAAAGCGTTTACCGGCGGTAGCGGCGAAGCGACCCTGACGAAAATTGGCGACGGCGTGTATAGCGCGAAAGGCACGTTTAGCGGTGTGGTGATTGATGGCGTGGAATATACCGGTACCTTTGAAGGCACCATTACCCTGGATAGCAACGGCA	MSGEPFRFTTSVDGTLDGTAFTSGTVDLSVSDVGDGKATITFDSGTFAGKAFTGGSGEATLTKIGDGVYSAKGTFSGVVIDGVEYTGTFEGTITLDSNGNVKSVKLTNITLTPAAGSGSHHWG

	ACGTGAAAAGCGTGAAACTGACCAACATCACCCTGACCCCGGCGGCGggttcccgagaccgtaatgc	STHHHHHH
n7_S10_nres10_C10	atactacggtctcaaggaATGCACATTACCGGCACCTTCACCTTTGATATCAACGTGAACGGCAAGAAATATAGCGGCAGCGGCGAATTTGTGACCGAAGAAGACGGCGTTACGTTTACCTTTAAAGGCACCACGGAAGATGGTAGCGTTATTGAAGGTACCGGCCGCCTGACCCGTCAGCCGGATGGCAGCATTACCTTAACGTTAACCGATATTAAAATTGATGGCAAAGCGGTTGGTGGTAGCGCGACTGGTCCGGCGACCTTACAGCCAGATGGTCATATTACCGCGACGGATATCACCGGCGAAATTACCGATggttcccgagaccgtaatgc	MSGMHITGTFTFDINVNGKKYSGSGEFVTEEDGVTFTFKGTTEDGSVIEGTGRLTRQPDGSITLTLTDIKIDGKAVGGSATGPATLQPDGHITATDITGEITDGSGSHHWGSTHHHHHH
n7_S10_nres10_D10	atactacggtctcaaggaGGTAAAGCGAAATTAGTGATTAACATTAAAGGTGGCGTGGGCGGCGAAGTTGAAGAACTGGTGATCGAAGGCGAAATCGATCCGGAAACCGGCGAATTTGAAGGCGAAGGCACCGCGAAATATAAAGACGGCACCACCGTGAAAGGCAAAGCGGAAGGCAAACTGACCTTTGAAGATGGTAAACTGGTTAGCTTTGAACTGGAAGGTGAACTGGATGATGGCAGCACCTTTAAATTGACGGGCACCGGCACGGGTACCTTACCAGCGGTGGGTGAAACCGGTACTATTGATGCAACCGCGACGGGCGAACTGACCCCGGCGCCGggttcccgagaccgtaatgc	MSGGKAKLVINIKGGVGGEVEELVIEGEIDPETGEFEGEGTAKYKDGTTVKGKAEGKLTFEDGKLVSFELEGELDDGSTFKLTGTGTGTLPAVGETGTIDATATGELTPAPGSGSHHWGSTHHHHHH
n7_S10_nres11_C11	atactacggtctcaaggaGCGAAAACCGAACACGTGAAATTTACCTTTAAATTCACCTTTACCCTGGATGGCGAAGAGAAAACCGGCACCGCGGAAGTGGATGCGGATCTGCCGACCGAAGTGGGTGGTAAAGGGAAAGGCAAAGGCGTTTTAAAAGACGAAAACGGTGAAAAGATTGGCGATTTCGAGGTGGACTTTGAACTGATCGGCGGCGATGGCGTGAAAGTGTATAAATTTGAATTCGAAGGCGAACTGAACGATGGTGCGAAAGGTAAAGGCAAGGGCACCGTGAAAACCAAACTGAAAGACGGCGAAAGCGGCGAAGTCGAAGCGGATGTGGAAGCCGATTTTAAGAAACATggttcccgagaccgtaatgc	MSGAKTEHVKFTFKFTFTLDGEEKTGTAEVDADLPTEVGGKGKGKGVLKDENGEKIGDFEVDFELIGGDGVKVYKFEFEGELNDGAKGKGKGTVKTKLKDGESGEVEADVEADFKKHGSGSHHWGSTHHHHHH
n7_S10_nres11_D11	atactacggtctcaaggaAGCAGCCGCGATGTGACCGTGAACTATACCATTAAAGGCACCATTCTGGGCAAACCGTTTGATGCGGGCAAAGTTACCGCGCCGGGCACCTTAAGCGATGGCGTGCTGACCATTAACAGCAAATATGTGGGTAAAGCGGGCGGTAAACCGGTGACCGATGTTACCTTTACCCTGGATCTGGGCTCTGATGAAGTGGGCTATAGCGGTAGCGGTACCGGCACCGCGGAAGGTACCTACGATGGCAAGAAAGTGGCGGGTAACGTGACCTGGAAAGGCAAAATCGTTGCGCCGACCCTGATTGAACTGGATATTACCATTGAAGATTGGAAAGAAGTGCCGGAAGCGggttcccgagaccgtaat	MSGSSRDVTVNYTIKGTILGKPFDAGKVTAPGTLSDGVLTINSKYVGKAGGKPVTDVTFTLDLGSDEVGYSGSGTGTAEGTYDGKKVAGNVTWKGKIVAPTLIELDITIEDWKEVPEAGSGSHHWGSTHHHHHH

	gc	
n7_S10_nres11_C12	atactacggtctcaaggaACCAAAACCGGCACCGGCGAAGGCGAATTCGAAATTAACGTGGATGGCGAGAAAGTGAAGGGCAAATTCGAGTTTGAAATTACCATCAATGACGATAACGGCAACGGTACCGTGAAAGGTAACCTGTATGATGATGAAGGCAAGAAAATTGGCAGCTTTGAAGGCCCGGTGAAAGTTACCGCGCAGGCGGATGGCACCTACGATGTGAAATTTACCGATCTGAAAATCGATATCAACGGCGAATATAGCGGCAAAGGTGAAGGCGAAGGTAAAACCAAAGAAGTGAACGGCCAGCTGGGCAAAATTGAAGGTGCGAAACTGAAAGGCGAAGCGAAGAAAATCCCGGGCGTGggttcccgagaccgtaatgc	MSGTKTGTGEGEFEINVDGEKVKGKFEFEITINDDNGNGTVKGNLYDDEGKKIGSFEGPVKVTAQADGTYDVKFTDLKIDINGEYSGKGEGEGKTKEVNGQLGKIEGAKLKGEAKKIPGVGSGSHHWGSTHHHHHH
n7_S10_nres11_D12	atactacggtctcaaggaGCGAAAACCCTGAAACTGGACGTGGATGTGGCGAAAGCGACCTACAACGGCAAAGAGTACGACAAAATGAAAGTTAAACTGACCATCGATAACCCGCCGACCAACGGCCAGAGCTTTACCGCGACCGGTGAACTGTATGATGCGAACGGTAACAAAATTGGCAACATTACCTTTAAAGGTACCGGCGATTTTACCAATGGCAAAACCGGCAGCGCCGAAGGTGAAGTGACCGACACCGATACAGGTGCGAAAGGCACCTTAAAACTGACCGATGTTAAAGTGAGCGGCGATACCGCCACGGCGACCGCGGATGAACTGGTGCTGAACAAACCAGGCAGCggttcccgagaccgtaatgc	MSGAKTLKLDVDVAKATYNGKEYDKMKVKLTIDNPPTNGQSFTATGELYDANGNKIGNITFKGTGDFTNGKTGSAEGEVTDTDTGAKGTLKLTDVKVSGDTATATADELVLNKPGSGSGSHHWGSTHHHHHH
n7_S10_nres12_E1	atactacggtctcaaggaATGACCTATAAAGTGAAACTGGATGTGAAATTTAAAAGCGAACTGGGCACCGGCAAATTTACCGCGGTGGTGGATCTGGATTTTGATGAAAGCACCGGTAACGGCAGCGGTACCATTACCGATGTGAGCGGCGATGTGGGTGGCGTGAAAATTAAAGGCGGCAAAGGCACCATTAAATTCGAAGGCGGTGAAAAGAAAGACGGCGTCTACTATGTGAAGAACATCAAAGTCAAATACACCGATGATAACGGCAACGGCTTTGAAGGTGAAGGCGAAGGGGAAATTAAACTGAACGATGATGGCACCGCGGAAGGTAAACTGGATATTGAAGGCAAAGTGATTCTGCCGAAACCGggttcccgagaccgtaatgc	MSGMTYKVKLDVKFKSELGTGKFTAVVDLDFDESTGNGSGTITDVSGDVGGVKIKGGKGTIKFEGGEKKDGVYYVKNIKVKYTDDNGNGFEGEGEGEIKLNDDGTAEGKLDIEGKVILPKPGSGSHHWGSTHHHHHH
n7_S10_nres12_F1	atactacggtctcaaggaATGACCAAAGTGCTTAAAGTGTATATGAAAGGCACCGACGAAGAAGGCCACGAGTTTGAAGTTGAAATGGAAATTGAAAACCCGAAAATCGATGAGGATGGCCGCTTTAGCGGCAAAGGCCCGGTGACCGTGACCATTAACGGCAAGAAATTTGAAGGCGAAGGGGAAGTCGAAGGCCGCATCGGCGAAGATGGCGTTACCGTGGAAGCGGAACTGCGTGCAGAAGGCACGGACGAAGACGGCACCAAATTCGAGGTTGAAGCGCGCGGTGAAGGCAAAGTGGAAGAA	MSGMTKVLKVYMKGTDEEGHEFEVEMEIENPKIDEDGRFSGKGPVTVTINGKKFEGEGEVEGRIGEDGVTVEAELRAEGTDEDGTKFEVEARGEGKVEEDENGVWNADLEATEAKVRIEP

	GATGAAAACGGCGTCTGGAACGCGGATCTGGAAGCGACCGAAGCCAAAGTTCGCATTGAACCGCCGAAACCGggttcccgagaccgtaatgc	PKPGSGSHHWGSTHHHHHH
n7_S10_nres12_E2	atactacggtctcaaggaATGAAAATGGATATCGATGTGACCATTAACCTGACCGATGAAAACGGCAAAGTTACCACCCTGAAAGCCAAAACCTATGAAGGCGAAGTGGATGTGGAGAAAGGTACCGTTAAATTTAAAGCGAAAATCTACGAAGGCACCGGCGGCAAATGGGAAGGTGAGTTCGAAGCGGAAGGCAAACTGACCAAACTGGCGGATAATAAATATCTGGTGCATTTCGAATTCGAAGGTGAATTTACCGATCCGAATGGCAACAAATATGAATTCAAGGGTGAAGGGGAAGGCGTTATCGAACAGAAAGGCGATACCTGGACCGGCGAACTGAAAGCAGAGGGCGAAGCGAAGAAACTGGAAggttcccgagaccgtaatgc	MSGMKMDIDVTINLTDENGKVTTLKAKTYEGEVDVEKGTVKFKAKIYEGTGGKWEGEFEAEGKLTKLADNKYLVHFEFEGEFTDPNGNKYEFKGEGEGVIEQKGDTWTGELKAEGEAKKLEGSGSHHWGSTHHHHHH
n7_S10_nres12_F2	atactacggtctcaaggaGCGAAAATTACCGGTACCCTGAACCTGACCGGCACCAGCAAAGATGGCACCAAATTTAGCATCAACGGCAACATTACCGACTTTACCTATGATAAAGAGACGGGCAAAATTAGCTTTTCGGGCACCATTACTGGTGGTACCGTGGGTGGTGTGACCATTACCGGCGGCACCTTTAGCGGCGAAGGCACTGCGAAACTGTTAGAAGATGGGAAAATCACCGATATTGATGTGGATGTGACGATTAAAACCGATAACGGCTATGTGCTGAAAGGCAAATTCACCGGTGAAGGCTATTACGATAAAGAAACCGGCCATCTGACCCTGAACTTAAGCGGCAGCGGCTTTGAACTGACCAAACCGGATCCGAACCTGggttcccgagaccgtaatgc	MSGAKITGTLNLTGTSKDGTKFSINGNITDFTYDKETGKISFSGTITGGTVGGVTITGGTFSGEGTAKLLEDGKITDIDVDVTIKTDNGYVLKGKFTGEGYYDKETGHLTLNLSGSGFELTKPDPNLGSGSHHWGSTHHHHHH
n7_S10_nres13_E3	atactacggtctcaaggaATGGTGACCGGCACCTTTGAAATGAACAACATTAAAGGCACCCTGGATGGCAAACCGTTTGAAGCGGAATATGCGAAAGGTACCCTGACCGATATCAAAATCGATTTTGAAAAGGGCATCATTACCGGCAAAGCGAACTTTACCATTCACTTTAAAGATGGCAGCAAACTGACCGTTACCGATGCGGATTTTAAAGCGACCGCGACCATTGATGAAGATGGTACCATTACCAACGGTAAAATTGAAGGCGAAGGCGAGGGCACCGATCAGAACGGCAAGAAATACAAAGTCAAATTTGAAGGTGACATCGTCGACGGCAAATATGACAAAGAGAAGAAACACCTGACCCTGAAAATTGACAACGTGAAAATGGAAATTACCAGCATTAAAggttcccgagaccgtaatgc	MSGMVTGTFEMNNIKGTLDGKPFEAEYAKGTLTDIKIDFEKGIITGKANFTIHFKDGSKLTVTDADFKATATIDEDGTITNGKIEGEGEGTDQNGKKYKVKFEGDIVDGKYDKEKKHLTLKIDNVKMEITSIKGSGSHHWGSTHHHHHH
n7_S10_nres14_F3	atactacggtctcaaggaGCGGAGAAAACCAAAACCGATGTTGATATTGATGTGACCGGCACCGCGGATGGCAAACCGTTTAAACTGAAAGTGAAACAGGAAAACGCGCCGACCACCGTGACCAAGAACGATAACGGCGTGTATACCTTTGAAATTGATGTGGATAACGTTACCG	MSGAEKTKTDVDIDVTGTADGKPFKLKVKQENAPTTVTKNDNGVYTFEIDVDNVTGEIGGKKVEGGKA

	GCGAAATTGGCGGCAAGAAAGTGGAAGGCGGCAAAGCGAAAATTAAAGGCAAATTCAAACTGACCGATCCGGAAGGTAAGAACGCGAAATTTGAGTTTGAAATCGAAATCGACGTGGAAGTGGATGGCGAGAAATATGGCGGCGTGGCGAAAGGTGAAGGCGAAGCCAAGACCAAAATGGAAGGTGATAAACTGGTGCTGACCGAACTGAAGGGTGAAGCCGAAGGCGAGCTGAAGAAAATTGAAGAAggttcccgagaccgtaatgc	KIKGKFKLTDPEGKNAKFEFEIEIDVEVDGEKYGGVAKGEGEAKTKMEGDKLVLTELKGEAEGELKKIEEGSGSHHWGSTHHHHHH
n7_S10_nres6_E4	AGGCTTGCCGAATatactacggtctcaaggaATGAAAACCCCGCTGAAAGTGACCTATAAAGGCAAAGAATATGACGGCGAACTGAGCGAAGTGGTGGATGGTAAAGCGAAAGTTACCGTTACCATTGATGGCAAAACCTATACCGGCACCGTGGAATGGGACGGCAACAAATTTGAAGGTAAACTGGATGAAACCGGCGCGAAAATTAAAGGCGAGCTGAAAGATGGCGTCGTTTATGCGGAAATTGATCCGGATGGCATTCCGGAAggttcccgagaccgtaatgcAAATTTCTGCTT	MSGMKTPLKVTYKGKEYDGELSEVVDGKAKVTVTIDGKTYTGTVEWDGNKFEGKLDETGAKIKGELKDGVVYAEIDPDGIPEGSGSHHWGSTHHHHHH
n7_S10_nres7_F4	atactacggtctcaaggaGGTGAAGCGTTTAAAGGCCTGGCGAACGGCACCATTGATGGCGTGCCGGTGACCAACTTACCGGTTGAAGGCAGCTTCGGCGATGGTACCATTACCATTTATGATCAGGATGGTAAGAAACTGGGCGAAGCGACCGTGAGCGGCAAAGATAACAAATTCAAGGGCGAAATTACCTATGACGGCAAGAAATACGAAGTTACCGGCGTGGTGAAGAAAACCGGCGAAGGCAAAGCGGAAGTGACCTTCGAAGCCAAAGAAGTGCCGGAAGAAggttcccgagaccgtaatgc	MSGGEAFKGLANGTIDGVPVTNLPVEGSFGDGTITIYDQDGKKLGEATVSGKDNKFKGEITYDGKKYEVTGVVKKTGEGKAEVTFEAKEVPEEGSGSHHWGSTHHHHHH
n7_S10_nres8_E5	atactacggtctcaaggaGCGCAGGCGTTTACCGTGACCGGCACTGTTGATGGTCAGACCTTTGATAGCGGCAGCGCGACGGGCACCTGGAGCGGTAACGAAGGTCAGTTAACCAGTGGTACCCTGGGTGGTGTGCCGTTCACCGGTGGCACGCTGAAAGGTACCGATATTGGCAACGGCAAATTTACCTTTACCATTACCGGCCTGACCATTAACGGCGTGCTGTATAGTGGCACCGGTACCGCGACCTTAAACAGCGATGGCGTGTGGACCCTGAACCTGAATGTGACCACCGTGGCGGCGggttcccgagaccgtaatgc	MSGAQAFTVTGTVDGQTFDSGSATGTWSGNEGQLTSGTLGGVPFTGGTLKGTDIGNGKFTFTITGLTINGVLYSGTGTATLNSDGVWTLNLNVTTVAAGSGSHHWGSTHHHHHH
n7_S10_nres8_F5	atactacggtctcaaggaGCGGAGAAACCGACCGTCGAAATTATTGGCACCGATGGCACCAAACTGACCATGGTGGTGACCGAAGGCGAACTGATTGGCGGCCGCGCGAAAGGTGAACTGTATCAGGATGGTAAGAAAGTGGGCGAAGGTACCCACGAAGTGAACGAAGATGCGACCACCGGCAGTGCGGAAGGCACCATTAACGGCAAACCGTTTCGCCATGAAGGTGAAGTGGTTGAAATTAGCCCGGATGGCAACAAAGTGGTGCTGGAAGGCCCGCTGGAAATTAAAggttcccgagaccgtaatgc	MSGAEKPTVEIIGTDGTKLTMVVTEGELIGGRAKGELYQDGKKVGEGTHEVNEDATTGSAEGTINGKPFRHEGEVVEISPDGNKVVLEGPLEIKGSGSHHWGSTHHHHHH

n7_S10_nres8_E6	atactacggtctcaaggaGCGCCGATTGCGGCGACCTTCACCGGCACCATTGACGGCAAAACCTTTACCAGCGGTACACTGACCGTTACCCGTGTGGGCACCACCGGTATTACCGGCGATGGCACGATTGATGGCGAGAAAGTGACCGGCTTTGTGTTTACCGGTGATGTGACGGGTGCGGGTACTCATAGCGGCACCGCGAGCATTGAAGAGAAAGGCTATACCGGTCCGGCCACCATTACCATTAGCGCGGATGGTACCACCGCGGATATTACGGCGGATCTGACCGCCGATAGCGCGggttcccgagaccgtaatgc	MSGAPIAATFTGTIDGKTFTSGTLTVTRVGTTGITGDGTIDGEKVTGFVFTGDVTGAGTHSGTASIEEKGYTGPATITISADGTTADITADLTADSAGSGSHHWGSTHHHHHH
n7_S10_nres9_F6	atactacggtctcaaggaACCATTACCGGCGATTTTGAACTGACCGCGGAAGGCAAAACCTATAAAGGTACCTTTACCATCAACGGCAGCGTGGAAGAAGGCGCGGAATTTGAAGGCACCCTGAAAGGCAACGACGGCACCATTAGCAGCGGCAAATTTACCGCCAAAGTGGGCAAAGATGGCAAAGCGAAAGTGAAATTCACCGATATTACCATTAACGGCGAGAAATATGCGGGCGAAGGCGAAATGCAGATTACCAACGAAAACGGTAAAACCACCGTGAAAATTAAAGGCGATCTGAAGAAAGTGGAAggttcccgagaccgtaatgc	MSGTITGDFELTAEGKTYKGTFTINGSVEEGAEFEGTLKGNDGTISSGKFTAKVGKDGKAKVKFTDITINGEKYAGEGEMQITNENGKTTVKIKGDLKKVEGSGSHHWGSTHHHHHH
n7_S12_nres11_E7	atactacggtctcaaggaAAGAAAATTCCGGAAATTATTGAAATTGATTTTACCATTAACGGCAAGAAATATCATATGACCATTGAACTGACCAAAGTGGAAGATCTGGGCGATGGCAAATATACCTTTACCGGCAAAATTAAAGATCTGACCGGCCATGGTGATGGCGAAGCGGAAGGCGAATTTGAATATGATGAAGAAGCGGGCGAAATTACCAAATTTAGCATTACCGGTGAAGTGGACGGCAAACTGTTTGATATTGAATTTAAACCGTATGATACCAAAGAATTTACGAAAGATGGTAAATATGGCCTGCGCTTTAAAGCGGATGCGAAAGGTGAATTTTATGATCCGggttcccgagaccgtaatgc	MSGKKIPEIIEIDFTINGKKYHMTIELTKVEDLGDGKYTFTGKIKDLTGHGDGEAEGEFEYDEEAGEITKFSITGEVDGKLFDIEFKPYDTKEFTKDGKYGLRFKADAKGEFYDPGSGSHHWGSTHHHHHH
n7_S12_nres12_F7	atactacggtctcaaggaATGGAAGTGAAAACCAGCAAAGCCAAACTGAACATTACCGGTGAACTGGATGGCAAAGAATTTAAACTGGAAGCCGAAGGCACCGTGAAATACATCCTGGAAGAAGAAAACGGCAAAGCGCATCTGAAATTCGAAATGGATTTTGAAGGCGAACTGAATGGTAAACCGGCGACCGGCACCCTGAAAGGCGATCTGGAAGGCGAAGTTCTGGGCAACGGCCTGTATGAATTAGAAGGCCCGTTTACCCTGACCGTGGATGGTGATGTGTATGAAGGTGAAGCGAAAGGCATTGTCAAATTTGATGAAAATGGCTATCTGACCGAAATGGATCTCGAAGTGACCGGCGCGAAACTGGTGAAGAAAGCGCCGGGCAGCggttcccgagaccgtaatgc	MSGMEVKTSKAKLNITGELDGKEFKLEAEGTVKYILEEENGKAHLKFEMDFEGELNGKPATGTLKGDLEGEVLGNGLYELEGPFTLTVDGDVYEGEAKGIVKFDENGYLTEMDLEVTGAKLVKKAPGSGSGSHHWGSTHHHHHH
n7_S12_nres12_E8	atactacggtctcaaggaACCAAAACGGTGCCGGTGAACCTGACCCTGAAAGGCGATGGCAAAACGCTGAAA	MSGTKTVPVNLTLKGDGKTLKLSGEFD

	CTGAGCGGCGAATTTGATGTGGAACTGCGCCAGGAAGGTGATGAAACCGTGCTGCGTGGTAAAGGTCCGGTGACCGGTACCTGGGAAGGCAAACCGGTTACCGGTGAAATGGAATTTGAAGCGCGCGGCAAACTGGATGGCGATAAAGGCGACTTCGATCTTGAAGGCCGCATGAAAATTGACGGCAAATTGTATAAATTTCGTGGCAAAGCGACCGTTACCCTGCATCGCGATGAAAACGGCATTACCGGCATTGATATTGATGCGACCGATGTGAAAATCGAAGAAATTGAAGAAggttcccgagaccgtaatgc	VELRQEGDETVLRGKGPVTGTWEGKPVTGEMEFEARGKLDGDKGDFDLEGRMKIDGKLYKFRGKATVTLHRDENGITGIDIDATDVKIEEIEEGSGSHHWGSTHHHHHH
n7_S12_nres8_F8	atactacggtctcaaggaGCGGCGAAAACCTTTAACGTGACCGGCACGTTTGATGGCGTGCCGATTGATCTGACCACCCTGGAATATACGATTGAAAACGGTAAACTGACCGTGAAAGGCAAACTGAACATTGGCGGCGTGGCGAAAGATTTTAAAGCGGAAGGCACCATCGAAGGCAACACCGCGACCCTGACGGGCACCTTAGATGGTGTTCCGGTTACCGCAACCCTGACCAACCTGGATATTAAAGATGATGAAATTCTGGGCACCGCCACCATTACCATGAAAACCGTGACCggttcccgagaccgtaatgc	MSGAAKTFNVTGTFDGVPIDLTTLEYTIENGKLTVKGKLNIGGVAKDFKAEGTIEGNTATLTGTLDGVPVTATLTNLDIKDDEILGTATITMKTVTGSGSHHWGSTHHHHHH
n7_S12_nres9_E9	atactacggtctcaaggaACCACCTTTAAAGGTCTGACCATTACCGGCACCCTGGATGGCGAACCGTTTACCCTGACCACCGATAAACCGCTGGATGTGGCGGGCAACAACAAACCGTTCGAAATTAGCGGCGATTTTACGCTGAACGGCAAACCGGTGGATGCGACGCTGACCGGTACCATTAACCTGACTGAAGGTAGCGCGAACACCATTCGCGGCACCATTGATGGCCGCCCGTTTGAATTGCGTGGTGGCACCGTGAGCATTGAAGATGGCGTGCTGCGCATCGATGGCATGGAAGGCCGCTTTCTGGATggttcccgagaccgtaatgc	MSGTTFKGLTITGTLDGEPFTLTTDKPLDVAGNNKPFEISGDFTLNGKPVDATLTGTINLTEGSANTIRGTIDGRPFELRGGTVSIEDGVLRIDGMEGRFLDGSGSHHWGSTHHHHHH
n7_S12_nres9_F9	atactacggtctcaaggaATGCAGACCACCATTAAAATTATTGGCGGCGAGCTGGATGGCGAACCAGTGGAAGGCGGTGAACTGGTGATTACCGAACGCGATGGCACCGAATTTACCGGCGAAGGCGAACTGGACGGCTTAGAACGTGGTCCGTTTCGTGGCCGTCATGATAAACTGGGTGAAGTGGGCGCGCGTGGTACCGCGGAAGGTGTGTTCGGTGGTTATCGTGTTCGCTTTGAATATGAAATTGTGGATGTGGCGGATGGTGTGATCACCGTTGAAATTACCGGTGGCACCCGCGAACCGGTGCCGGCGggttcccgagaccgtaatgc	MSGMQTTIKIIGGELDGEPVEGGELVITERDGTEFTGEGELDGLERGPFRGRHDKLGEVGARGTAEGVFGGYRVRFEYEIVDVADGVITVEITGGTREPVPAGSGSHHWGSTHHHHHH
n7_S12_nres9_E10	atactacggtctcaaggaAGCAACACCGTCACCGGCAACCTGACCCTGACCGTGGATGGTGAAACCATGACCGGCACCGTGACCATGGATGCAGCGAATGCGGAAAAGGCGGGCGCAACTTTTACCTTCACGGGCGTGGTGTATCGCGGCAAACCGGTTAGTGGCACTGGC	MSGSNTVTGNLTLTVDGETMTGTVTMDAANAEKAGATFTFTGVVYRGKPVSGTGTLNFNAGEKGSWT

	ACCCTGAACTTTAACGCGGGCGAAAAGGGCAGCTGGACCGTTACCTTTGAAGGCTATGAAGTTAAAGGCGAAGGCTTTATGAGCAAAGTGGATAGCAACAACAAATTTGATCTGGATCTGAAAGGTGGCGAACGCGTGCCGCTGCCGggttcccgagaccgtaatgc	VTFEGYEVKGEGFMSKVDSNNKFDLDLKGGERVPLPGSGSHHWGSTHHHHHH
n7_S12_nres9_F10	atactacggtctcaaggaGCGAACGCGACCGGCACCTTTAAAGCGGATCTGGATGGCACCGTGGTTACCGGTACCTTCACCGTTACCAACCAGACTGCGGGCGGCAATGGCATCACGGGCACCCTGTATAACATTACGGGCCATGCGGATGGTACGTTTACCATTACCAATCTGACCAACGGCAAAGGTACCGTGACCCTGACCATTGATGGCGTGACCTATACCGGCAACGTGACCGTGGATAGCACCATCACCGCGGGCTCTACGGGTACTCTGACCTTTACCGATCTGAAACGCGTGGCGGCGGCGCCGggttcccgagaccgtaatgc	MSGANATGTFKADLDGTVVTGTFTVTNQTAGGNGITGTLYNITGHADGTFTITNLTNGKGTVTLTIDGVTYTGNVTVDSTITAGSTGTLTFTDLKRVAAAPGSGSHHWGSTHHHHHH
n7_S14_nres9_E11	atactacggtctcaaggaGCGACCCAGACCATTGTGGTGACCACCCCGGATGGCGAAACCCATACCCTGCAGACCAACCTGGCTCTGAAACCGGGTGATACTGGTGGCACCGGTACTTTCTCTGGCACTGTGGCGGGTCGTGCGATTAGCGGTGGTACCCTGACCTTTGAACGTCTGGAACCGGGCGCGCGTTTTACCTTAACCGATGATGCGGGCAACGTGATTACCGGCCGTGTTGTGCGCGTTGAAGAAGGTGGCGCGATTCATCTGCAGGCGGATGCGGTGACGTTTGTGGATggttcccgagaccgtaatgc	MSGATQTIVVTTPDGETHTLQTNLALKPGDTGGTGTFSGTVAGRAISGGTLTFERLEPGARFTLTDDAGNVITGRVVRVEEGGAIHLQADAVTFVDGSGSHHWGSTHHHHHH
n7_S14_nres9_F11	atactacggtctcaaggaATGGAGAAATTTAGCGGCGAAATTCGCATTCTGGATGAAAACGGCGAAGTTGTCTTTGAAGGCCGCTTCGAAGGTGAACAAGTTGGCGAAAACGAACAGGTTCTGCGCGCCGAAGGCGAATTTCGTGGCGAACCGTTTGCGTTTGAAGCGCGCGTGGAAGGCAAACTGGAACCGGGCAAAGAATTCGAAGCGGAAGGTACCTTAGGCGGTCGTCGCGTTGCGTTACGCCTGCGTGTGTTAGAATTTGGCGATGGCAAATTAGTGGTGCAGCTGCTGGAATTCCGTGAAGTGGCGGCGggttcccgagaccgtaatgc	MSGMEKFSGEIRILDENGEVVFEGRFEGEQVGENEQVLRAEGEFRGEPFAFEARVEGKLEPGKEFEAEGTLGGRRVALRLRVLEFGDGKLVVQLLEFREVAAGSGSHHWGSTHHHHHH
n7_S8_nres10_E12	atactacggtctcaaggaAAAGTTAAAGCGACCGGCGTGATTCCGGTGCGTGGTGAACTGAGCGGCGTTCCATTTGAAGGTGAAATGAAAGTGGATTTCGATTTTGAAGCGGGCAAAAGCGGCGAATTTGATTTTACCCTGACCCTGGATATTGATGGTAAAACCCATGTGGCGGTGGGCAAAGGCAAATACGATGCGATTAACGAGGGCGAAACCGGTAAAGGCGAGTTCGGCTTTTATGATAAAGATACGGGCGTTACCGGCGTTGGCGAAGTGAAATTTACCGTGAAAGATGGCAAAATCACCGGCGAAGCGCGCGCGGAAGGCGAAATTCCGAAGAAAg	MSGKVKATGVIPVRGELSGVPFEGEMKVDFDFEAGKSGEFDFTLTLDIDGKTHVAVGKGKYDAINEGETGKGEFGFYDKDTGVTGVGEVKFTVKDGKITGEARAEGEIPKKGSGSHHWGSTHHHHHH

	gttcccgagaccgtaatgc	
n7_S8_nres11_F12	atactacggtctcaaggaATGACCGTGACCGTTGAAGCG GAAGTGACCGGCAACATTGGCGGCGAGAAATTTA AAGGCGAGGCGAAAATCACCCTGAAAATTACCGA AAAGGATGGCGTTCTGACCTTTGAAGGGGAAGG CGAAGTTACCCTGCGCTTTGAAAACGGCCTGGTT CTGAAAGGCAAAATTATTGATCTGAAGGGTGTGAT TAAAGAAGACGGCAGCTTCAAAGGCAAAGGTAAA TTCGAAGGTGAAAACGATGGCATTAAAGCGAAGG GCGAAGCGTATATTGAAGGCAAGCTGGTTGATGG CAAACTGGTGGATGTGAAAGTGAAAATCGATGGC GAACTGGAGAAAGTGGAAGAAggttcccgagaccgtaat gc	MSGMTVTVEAEVT GNIGGEKFKGEAKI TLKITEKDGVLTFEG EGEVTLRFENGLVL KGKIIDLKGVIKEDG SFKGKGKFEGEND GIKAKGEAYIEGKLV DGKLVDVKVKIDGE LEKVEEGSGSHHW GSTHHHHHH
n7_S8_nres11_G1	atactacggtctcaaggaATGAAAGTTGAACTGGATGCG GAAGCGGATCTGAAAGACGAAAATGGCAACCTG GTGAGCAAATTTAAGTTTAAACTGGAAGTGGAAAT TGATGAGAACGGTGAAGTGACCGGCGAAGCGGA ATTTAAAGATCAGGGCGGCATCAAAGAAATGAAG ATGGAAGTGAAAGGCAAATATGATAAAGAAACCG GCAAAATTACCGGGGAAGTCGAAGGTGAATTTGT GGATGAAAACGGCAAGAAACATAAATTCAAAGGC GAATTTGAAGGCAAAGTGGATCTGGAGAAAGGTA AAGGCACCATGAAAATGGACCTGACCCTGCTGC CGGAAGAGAAAggttcccgagaccgtaatgc	MSGMKVELDAEAD LKDENGNLVSKFKF KLEVEIDENGEVTG EAEFKDQGGIKEM KMEVKGKYDKETG KITGEVEGEFVDEN GKKHKFKGEFEGK VDLEKGKGTMKMD LTLLPEEKGSGSHH WGSTHHHHHH
n7_S8_nres12_H1	atactacggtctcaaggaATGAAACTGAAATTGAAAGCGA TTGCGGATTTTGATCTGGTGGATGAAGATGGCAA GAAAATTGGTACCATGAAAGCCGAATTCGAAGCG GATGTGCTGGAAGGCGGTAAACTGGAGAAAGCG CCGTTTACCTTTCGCGGCGAAATTAACGGTCAGG ATGTTAGCGGTACCGGTGAAGCGCAGGGCCGCG TGGAATTAGAAGAAGGTGGCAAAGTTAAAGCGGA AGTGGATTTTAAAATCACCGAAAACAACCTGGGC TTTCAGGGTGAAGGCACCGCGCGTGGCGAAGG CAAATTGGAAGATGGTGTCGCGACCGTGGATATG GAAGGCACCATTACCGGCAAACTGCCGGAAggttc ccgagaccgtaatgc	MSGMKLKLKAIADF DLVDEDGKKIGTMK AEFEADVLEGGKLE KAPFTFRGEINGQD VSGTGEAQGRVEL EEGGKVKAEVDFKI TENNLGFQGEGTA RGEGKLEDGVATV DMEGTITGKLPEGS GSHHWGSTHHHH HH
n7_S8_nres13_G2	atactacggtctcaaggaAGCACCAAATATACCGGCAAAT TTGAAGCGACCGTGACCGGTACCATTGATGGTAA ACCGTTTAAAGCGAAACTGAGCGCGGAATATGAA GCAGAAGTGGATAACCCGGAAGACGGCAAACCG TTCACCTTTCGTGGCCGCTTTCGCGGCGAACTG ACCTTTGATGATGGCACCGTGGTGCCGGTGGAA GGCGAATTCGAAGGCAAAGCGGAAGGTCCGCTG AGCGATTTCACCGGCGAAGTGAAAGTGTATATCA AAATGAAAATCGATGGCAAACTGTACACCGGCAG	MSGSTKYTGKFEA TVTGTIDGKPFKAK LSAEYEAEVDNPE DGKPFTFRGRFRG ELTFDDGTVVPVEG EFEGKAEGPLSDFT GEVKVYIKMKIDGK LYTGSGTTKFTGKI DTEKKVLDLDLGTI

	CGGCACCACCAAATTTACCGGTAAAATTGATACC GAAAAGAAAGTGCTGGATCTGGATCTGGGCACC ATTACCGTGGATTTTACCGAAGCCGAAGAAggttccc gagaccgtaatgc	TVDFTEAEEGSGS HHWGSTHHHHHH
n7_S8_nres6_H2	ACTGCCCatactacggtctcaaggaACCGAAGAACGCGT TCGCGGTGAACTGGATGGCGTTCCGTTCGAAGG TGTTCTGCGCGGTCCAGAAGAAGGTCGCGTCCG CATTGAAGGCGAAGTGGATGGTCAGCCATTCGAA GCCGAGGGCGAACGCGAAGGCGATCGTATTCGC CGCTTTCGTGGTGAATTAGATGGCCGCGAATTTG AAGGTGAAGGTCGTCGCGTTGCGCCGGGTTTAT GGGAAGCGCGTGCAGAAACCTTGCCGGTGCTG GAAggttcccgagaccgtaatgcTACCGG	MSGTEERVRGELD GVPFEGVLRGPEE GRVRIEGEVDGQP FEAEGEREGDRIR RFRGELDGREFEG EGRRVAPGLWEAR AETLPVLEGSGSH HWGSTHHHHHH
n7_S8_nres8_G3	atactacggtctcaaggaATGATGAAAGTTAAAGTCGATG TGGAAGGCACCCTGGGCGGCAAATATAAAGTGAA AGCGGTGTTAGAAGGCAACGACGATGGCAGTAA AGCGCGTGGCGAGGGCGAAATCGAAGGCGGCA CCAAAATTGAAGTTGAGGGCACCATTAAAGATGG TAAAGTTACCGACATCAAAGCCGAAGCGGAACTG CCGGATGGCAACAAAATTCTGGGTGAGGGTGAA GGCGAATACGATGAAAACGGCAAAGGCAAAGCG ACCCTGGAAGGTGAAGTTATTAAGAAAGAAAAGg gttcccgagaccgtaatgc	MSGMMKVKVDVE GTLGGKYKVKAVLE GNDDGSKARGEGE IEGGTKIEVEGTIKD GKVTDIKAEAELPD GNKILGEGEGEYD ENGKGKATLEGEVI KKEKGSGSHHWG STHHHHHH
n7_S8_nres9_H3	atactacggtctcaaggaATGCCGAAAACTGTTCGCGTG CGTGTGCGCGGCGAAGTGGATGGCGTGGAAATT GAAGGGGAAGTGGTTCTGGAAGGCGAAGAAATT GACGGCGTCTTTGTGGGCGAAGGTGAGGGAGAA GGCGAAACCGCAGATGGTCGCCGTTTCCGTGGT CGTTTGCGCGCGACCTTACCACGTGGTGAAGTG GGTGAAGGTGAACTGGAAGCGGAATTCGAAGAT GGCACCCGCTTTCGCGGCCGCGTTCGTGGTCAG GTTGATGGTGATGAAGGCGAGGGCGAAATCGTT GGCGGTGAATTTATTCCGCCGAAAggttcccgagaccg taatgc	MSGMPKTVRVRVR GEVDGVEIEGEVVL EGEEIDGVFVGEG EGEGETADGRRFR GRLRATLPRGEVG EGELEAEFEDGTR FRGRVRGQVDGDE GEGEIVGGEFIPPK GSGSHHWGSTHH HHHH
n8_S10_nres7_G4	atactacggtctcaaggaATGAAGAAATACAAATTCACCA TTACCCTGAACGATGGCACCAAATATACCGGCGA AATGGAAGTGAACGAAGATGGCGTGGTTCGCTTT GAAGGTGAGGGCGAAGACGGTACCAAAGTGGTT GGCGAAGGCGAAGTGGATCTGGAAACCGGTGAA GGCACCGCGGAACTGGAAGCGGATGGCGGCAA AACCAAATGGAAAGCGACCTTTAACCTGAAGAAG GGCACCGGCGAGATCTATGATGAAAACGGCAACA AAGTTGGCACCGTGAAAATTGAAGAAGTTGAAGT CGAAGAAggttcccgagaccgtaatgc	MSGMKKYKFTITLN DGTKYTGEMEVNE DGVVRFEGEGEDG TKVVGEGEVDLET GEGTAELEADGGK TKWKATFNLKKGT GEIYDENGNKVGT VKIEEVEVEEGSGS HHWGSTHHHHHH
n8_S10_nres8_H4	atactacggtctcaaggaGAAAAGAAAACCCTGAAAGGC	MSGEKKTLKGTFK

	ACCTTTAAAAGCAGCGACGGCAAACTGAGCGGC<br>GAAATTACCCTGGAAGGCGATTTTCAACCGGGCG<br>GTAAAGCGACCTTTGAAGCGAAAGGCGAACTGG<br>AAGGTCAGCCGGTTACCGTGAAAGGTGAGGCGA<br>AAGTGGGTGAAGGCGGTCGCCTGTCTGGCGAAG<br>GTGAAGCAGATGTTGGTGGCAAGAAATATCCGGC<br>GACCCTGGATGTGGGTGCGGATGGCAAAGGTAC<br>GCTGAAAATTGGCGAGGGCGGCAACGTTTATACC<br>GCGGATGTGACGCTGGATGAAAAGATTGAACTGA<br>AACCGCCGGGCGAAggttcccgagaccgtaatgc	SSDGKLSGEITLEG<br>DFQPGGKATFEAK<br>GELEGQPVTVKGE<br>AKVGEGGRLSGEG<br>EADVGGKKYPATLD<br>VGADGKGTLKIGE<br>GGNVYTADVTLDE<br>KIELKPPGEGSGSH<br>HWGSTHHHHHH
n8_S10_nres8_G5	atactacggtctcaaggaATGTATTATAAAGGTACCTTCGA<br>GGGTGAAATCGATGGCGTGAAATTTAAAGGCGAA<br>TTCGTTGTGGACCTGGACAACAACTATTTTCAGAT<br>GAAAGCGGTGGACGAAGATGGCAAACTGCTGAA<br>AGCGAAAGGCGAGATCGATCTGGAAAACGGCAC<br>CTTTAAATTTGAAGCGGAATATGATGGTGGCAAAG<br>GCGAAGGGGAAGGCGAGGGCGATCTGACCAAA<br>GTTGGCGGCAAATTCAAATTCGAAGGTGATATCG<br>GCGGTAAGAAAGTGAAAGGAGAAGGCGAAATTA<br>CCGAAGAAATTGACGAGTTTGAAGAACTGTTTggtt<br>cccgagaccgtaatgc	MSGMYYKGTFEGE<br>IDGVKFKGEFVVDL<br>DNNYFQMKAVDED<br>GKLLKAKGEIDLEN<br>GTFKFEAEYDGGK<br>GEGEGEGDLTKVG<br>GKFKFEGDIGGKKV<br>KGEGEITEEIDEFE<br>ELFGSGSHHWGST<br>HHHHHH
n8_S10_nres8_H5	atactacggtctcaaggaGCGAAAATTGTGAAAGGTGAG<br>TTCGAATTGGAAAACGGCACCAAAGGCAAGTTCG<br>AGGGTGAAGTGAAAGACGGCAAAGGCGAATTCG<br>AGGGCGAAATCGATAAAGATGGTGTTAAAGGTGT<br>GTTTAAGGGCGAGGTGAAACTGGATGAAGATGG<br>CAAAATTTGGGGTGAATTTGAAGGTGAACTGGGT<br>GGCAAAGATTTCAAAGGTAAATTCGAAGGCAACG<br>TGAAAGGCGATGGTAAAGGCACCATCGAACTGG<br>ACATCGATGGCGAGAAAATTAAAGGCGAAATGTA<br>CGTTAAAGTGGAAGATGCGCCGAAACCGGTGCC<br>Gggttcccgagaccgtaatgc	MSGAKIVKGEFELE<br>NGTKGKFEGEVKD<br>GKGEFEGEIDKDG<br>VKGVFKGEVKLDE<br>DGKIWGEFEGELG<br>GKDFKGKFEGNVK<br>GDGKGTIELDIDGE<br>KIKGEMYVKVEDAP<br>KPVPGSGSHHWG<br>STHHHHHH
n8_S10_nres8_G6	atactacggtctcaaggaAAGAAAATCACCGGCGGCAAA<br>TTTGAAGGCGAAGTGGGTGGCAAGAAAGTGGTG<br>GTCGATATTACCGATGTGAAAGATCTGGGCGATG<br>GCAAATATGAAGCGACGTTTAAAGCGACCGGCCT<br>GGGCGGTGATGGTAAACTGACCTTTAAACTGTTT<br>GAAGATGGCAGCATCAAAGGTGAATTCACCGTGG<br>ATGCGAACGGTAAGAAAGGCACCTTCGAAGGTAA<br>AATCGATGAAAACGGCGAAGGTGAAGTGGAACT<br>GGATTTTGATGGCCAGAAAGGTAAAGGCAAAATT<br>ACCCTGACCCTGGAAGAAggttcccgagaccgtaatgc	MSGKKITGGKFEG<br>EVGGKKVVVDITDV<br>KDLGDGKYEATFKA<br>TGLGGDGKLTFKLF<br>EDGSIKGEFTVDAN<br>GKKGTFEGKIDEN<br>GEGEVELDFDGQK<br>GKGKITLTLEEGSG<br>SHHWGSTHHHHH<br>H
n8_S12_nres6_H6	atactacggtctcaaggaACCACCGTGAAAGTGACCGGC<br>ACGATTGGCGGTCAGGCGGTGGATCTGACCGGT<br>ACTGAAATTGCGCCGGGCGTGTATCAGGTGGAA	MSGTTVKVTGTIGG<br>QAVDLTGTEIAPGV<br>YQVEGTIGGKAFS

	GGCACCATCGGTGGTAAAGCGTTTAGCGGCACC CTGACCGCGGCGAATGGTACCTGGAGCCTGAAA GGTACTCTGGATGGCAAACCGTATGATGCGGGCA GCGGTACACTGAGTGCTGGCGGTGCGTTTTCTG GCACTGATGCAACTACCGGTGCGGCAGTTTCTG GTACCATTACCGCGACGGCGGCGggttcccgagaccgt aatgc	GTLTAANGTWSLK GTLDGKPYDAGSG TLSAGGAFSGTDAT TGAAVSGTITATAA GSGSHHWGSTHH HHHH
n8_S12_nres8_G7	atactacggtctcaaggaAAGAAATACACCTTTAAAGCGG AAATTGATATCAACGGCGAGAAACATGTGCTGAC CGGCGAACTGGAAAAGGAAGGCAACACCTATAG CGGCAAAATTACCGATGGTACCATCTTTGGCAAA CCGTTTACCGGTACCTTTGAAAACGTGGAAATTG GCGGTCCGGGTAAATGGTTCCCGGGTACCCTGG GTGGTAAACCGATGCGCTTTAAAATCGCGATCGA TGAACTGGATGAAACCGGCACCATTCATTTTGAAT TGGAAAACGGCCTGAAAGGCGAAGGCACGATTA CCATCACCGAAGTGCCGCCGggttcccgagaccgtaatg c	MSGKKYTFKAEIDI NGEKHVLTGELEKE GNTYSGKITDGTIF GKPFTGTFENVEIG GPGKWFPGTLGGK PMRFKIAIDELDET GTIHFELENGLKGE GTITITEVPPGSGS HHWGSTHHHHHH
n8_S12_nres8_H7	atactacggtctcaaggaACCCGCTACTATGTGAAATTTG AATTTGAAGCGGATGGTAAGAAATTCGTGGTCGA AGGTGATGCGGGCAAACTGGGCGAATGGGGCCC AGCGACTATTACCGTCGATGGCAAAGCGTATCCG GGCGAAATTAAAATTACCGCGATTGATGGCGATAA AGTGGTGGTGGAATTCAAAGGCGAGATCGACGG CAAACCGATTGAAGGCAAAGGTACCTTTACCGTG AAAGATGGTAAAGGTGAAGGTAAAGTGGAAATGG AACTGGATGGCAAGAAACTGGAAGGCGAAGGCA CCATGGAACTGAAAGAAGTGggttcccgagaccgtaatgc	MSGTRYYVKFEFE ADGKKFVVEGDAG KLGEWGPATITVDG KAYPGEIKITAIDGD KVVVEFKGEIDGKP IEGKGTFTVKDGKG EGKVEMELDGKKL EGEGTMELKEVGS GSHHWGSTHHHH HH
n8_S14_nres8_G8	atactacggtctcaaggaACCAAAACCTTTGAAGTGTATG ATGCGAACGGCAACAAAATTGATACCTTTACCGT GGTGTATCGCGATGGCAAAACCATTGTTTGGGTG GATAGCAACGGCAATAAAATTGTGTGGGAACGCG TGGGCGATGATAAGAACCGCATTACCGCGACCGA TGAAAACGGTAAAGTTACCAACTGGACCATTCTG GAATTTGAAAATGGCGAATTACTGGTGGTGAAGA ACGAAGAAACCGGCGAAGTGATTACCTTCAAAGG CGATACCGGCTGGAGCGAAGAAGGCTTTggttcccg agaccgtaatgc	MSGTKTFEVYDAN GNKIDTFTVVYRDG KTIVWVDSNGNKIV WERVGDDKNRITAT DENGKVTNWTILEF ENGELLVVKNEETG EVITFKGDTGWSEE GFGSGSHHWGST HHHHHH
n8_S8_nres11_H8	atactacggtctcaaggaAGCAAAATCCGCATCTACAAG GGCAAGTTTAAATTCAAGAACAAGAAAACCGGCG AAACGGGTGAAGGCGAGGCGGAAGCGGAAATC GATGAAAGCAACGATTACAAAGGCACCTTCAAATT TGAAGCGAAGAACGATGATACCGGCGAGAAATTT AAAGGGGAAGGGAAAGTTAAACTGGATCCGGAA ACCGGTGAAGTGACCCTGACCGATCTGGAAGTG	MSGSKIRIYKGKFK FKNKKTGETGEGE AEAEIDESNDYKGT FKFEAKNDDTGEK FKGEGKVKLDPET GEVTLTDLEVENLE TGVKGKGEGKGGK

	GAAAACCTGGAAACAGGCGTTAAAGGCAAGGGC<br>GAAGGCAAAGGCGGCAAAATTAAAGGCGAAGAG<br>GGCTTCGATTTCAAGTTTGAAATCAAACTGGATAA<br>CGGTGAAACCCTGGTGGCCGAAGGTAAACTGAA<br>ACTGGTGGAAGTGCGCGAAggttcccgagaccgtaatgc	IKGEEGFDFKFEIKL<br>DNGETLVAEGKLKL<br>VEVREGSGSHHW<br>GSTHHHHHH
n8_S8_nres12_G9	atactacggtctcaaggaATGCATCTGAAAGGCGAGGGC<br>AAAGTGAAACTGGACGGCGATGGTGATGAATTTA<br>AAGGTGAAGGTAAAGCGTATGTGGATAGCGATCT<br>GGATGAAAACGGCAACGGCTTCAAACTGAAATTC<br>GAAGGGGAAGTTAAATTCAAAGATGGCACCGTTA<br>ACAAATTCGTGGGCGAAACCAACGATGGCGTGC<br>TTAAAATTGAAGATGGTAAAGGCAAACTGCAGCT<br>GCCGGTGAAATTTCGCTTTCTGGATAAAGGCGGC<br>GTGGGTGAAGCGAAATTTGATGGCGGTGAAGTG<br>ACCGTGGAAGACGGCAAATTTAAACTGGATGCCG<br>AAGCGGATTTTGAAGCCGAACTGGATGATGGTAC<br>CAAATTCAGCGGCAAAATGAAATTGGATGTGGAA<br>GGCGAAGTCGAAGAAGTGGAAggttcccgagaccgtaat<br>gc	MSGMHLKGEGKVK<br>LDGDGDEFKGEGK<br>AYVDSDLDENGNG<br>FKLKFEGEVKFKDG<br>TVNKFVGETNDGV<br>LKIEDGKGKLQLPV<br>KFRFLDKGGVGEA<br>KFDGGEVTVEDGK<br>FKLDAEADFEAELD<br>DGTKFSGKMKLDV<br>EGEVEEVEGSGSH<br>HWGSTHHHHHH
n8_S8_nres12_H9	atactacggtctcaaggaGCGAACAAAGTGCTGGAAGCC<br>GAAGGCACCGTGAAATTTGAAGGCGATCTGAAC<br>GGCCAGCATTTTAGCGGCGAGGGCAAAGCGAAA<br>GCGATTTACGATGAAGGCAACAAAGAACTGAAAG<br>TAGAATTCGAAGCTGAAGGTAAACTGAATGGCCA<br>GGATATTGGTATGAAAGGCGAAGGTACCATCAAA<br>GGTGTGGATCTGAGCAAGGGCTATTTTAAAGGCA<br>AAATGGACATGAAAGTGGATATTACCGGCCTGGG<br>CGGTAAAGGTGAAGTGACCGAAGCAGAATTAAAA<br>GATAACGGCGGCAAAGGTTGGGTGATTGAAGCG<br>GAAGGTGAAGCGCAGACCCCGCTGGGCAACTTC<br>AAGTTCAAAATCGAGCTGGACCTGGACAAGTTTA<br>AAGAGATTAAAggttcccgagaccgtaatgc	MSGANKVLEAEGT<br>VKFEGDLNGQHFS<br>GEGKAKAIYDEGN<br>KELKVEFEAEGKLN<br>GQDIGMKGEGTIK<br>GVDLSKGYFKGKM<br>DMKVDITGLGGKG<br>EVTEAELKDNGGK<br>GWVIEAEGEAQTP<br>LGNFKFKIELDLDK<br>FKEIKGSGSHHWG<br>STHHHHHH
n8_S8_nres6_G10	atactacggtctcaaggaAGCAAGAAAGTGCTGTTCGAA<br>GGCGAAGTGGATGGTGTGCCGTTTACCGGCGAA<br>TTTGATGTTGTTCCGGATGGCGCGCCAGCGACC<br>GCCACCATTAACCTGAATGGCAAAGATCATGTGTT<br>CACCGGCACCTTTAGCAACAACACCTTTGTGGGC<br>ACCGATGCGGCGACCGGCTGGAAACTGACCGTT<br>AAAATTGATGGCGATACCGCGACCCTGACCCTGG<br>ATAAGAACGGCAAGAAATATCAGGCCGAAGGCAA<br>AGTGGTTGAACCGCCGggttcccgagaccgtaatgc	MSGSKKVLFEGEV<br>DGVPFTGEFDVVP<br>DGAPATATINLNGK<br>DHVFTGTFSNNTFV<br>GTDAATGWKLTVKI<br>DGDTATLTLDKNGK<br>KYQAEGKVVEPPG<br>SGSHHWGSTHHH<br>HHH
n8_S8_nres6_H10	CTACTatactacggtctcaaggaGCGAAGAAAGTGGAATT<br>TAGCCTGACCGATGCGAACGGCAATACCATTCAG<br>GGCACCGCGGAAGAAGGCAAAGAACTGCCGGTT<br>TATGATCAGAATGGCAACCTGGTGGGTACCGCGT	MSGAKKVEFSLTDA<br>NGNTIQGTAEEGKE<br>LPVYDQNGNLVGT<br>AFIKDGELYFKDNN

	TTATTAAAGATGGCGAGCTGTATTTCAAGGATAACAACGGTAACGTGTATAAAATCGAAGTGGACGGCGATAAAGCGACCGCGACCATTAACGGCGTGGAATATAGCGGCACCGTGAAAGAAATTGAAGCGCCGggttcccgagaccgtaatgcGTCAT	GNVYKIEVDGDKATATINGVEYSGTVKEIEAPGSGSHHWGSTHHHHHH
n8_S8_nres6_G11	atactacggtctcaaggaACCTATACCGTGACCGGCGAATGGAGCGATGGTAAGAAAACCAGCGGCACCCTGACCCTGAGCCCGGGCAACAAATTTACGCTGACCTTTGATGATGGTCTGGTTCTGACCGGTAATTGGCCGGCGGCAGGTCAGCCGTTTAGCGGCAAAGATCAGAACGGTAACCCGTTTACCGGTACCAGCGCGGCGGCGGGTCAGGAATTTACCATTACCATCGATGATAACGGCAAGAAAACCGTGTTTAAAGGCCTGCTGACCGCGGATCCGGATAGCggttcccgagaccgtaatgc	MSGTYTVTGEWSDGKKTSGTLTLSPGNKFTLTFDDGLVLTGNWPAAGQPFSGKDQNGNPFTGTSAAAGQEFTITIDDNGKKTVFKGLLTADPDSGSGSHHWGSTHHHHHH
n8_S8_nres6_H11	AatactacggtctcaaggaGCGATTGTGGGCGAAGCGAAGAACAAAGACGGCAACACCGCGAAATTTCGCATTGAAGATGGCAAAGTCAAAGGCGAAGTGACCGTGAACGGTAAAACCTATGAATTTGAAGCGGAACTGATTGATGGCACCAAATTCGAAGGCAAAATTAAAGGCGCGGACGGCACCGTGAGCGGCGAAATCGAAGATGGTAAAGTTAAAGTGGAAGTGGATCTGAACGGCAAAACGGTGAAACTGGAAGGCACCTACGAGAAAGTTGAAggttcccgagaccgtaatgc	MSGAIVGEAKNKDGNTAKFRIEDGKVKGEVTVNGKTYEFEAELIDGTKFEGKIKGADGTVSGEIEDGKVKVEVDLNGKTVKLEGTYEKVEGSGSHHWGSTHHHHHH
n9_S10_nres6_G12	atactacggtctcaaggaAAACCGACCGTGACCTTTAAAGCGGATGATGGCACCACCGGTAAAGGCGAAGTGGAGATTGGCGATAAAATTGAATTTAAAGGTGAACTGAGCGATGGCAATACCTTCAAAGGCGTGATCGATCCGAAAGCGGGCACCTTTACCGCGCAGAACACCACTACCGGTCTGACCGCGAAAGGCAAAGTCGAAGGCAACACGTTTAGCGGCACGTTTGATGACGGCACCAAATTCAAGGGCGAAATCAAAGAAGATGGCAACGTTTATATTTATGCGCAGACCGCGCCGAAAggttcccgagaccgtaatgc	MSGKPTVTFKADDGTTGKGEVEIGDKIEFKGELSDGNTFKGVIDPKAGTFTAQNTTTGLTAKGKVEGNTFSGTFDDGTKFKGEIKEDGNVYIYAQTAPKGSGSHHWGSTHHHHHH
n9_S10_nres6_H12	atactacggtctcaaggaACCATTACCTTTAAAGGCACCGTGCTGGGCAAAGAAATTACCGGCACTGTGACTGGCACTCCTGGCACCGGTACTGCGACCGCGGATTTAGGCGGTGAACATTTTACGGGTACCATTAGCGCGGGCCCGCTGACCGTTACCCTGACCAACGCCAAAGGTGATGTGATTACCATCAAAGTTGATGGCACCAAAGTGACCGTGAGCGGCGATGTGGATGGCAAGAAATTTGAAGGCGAAGGCACGTATACCCCGGGTAGCGATCATATTGATCTGGGCGTGGCGGAAGAAATCCCGGGCGTGggttcccgagaccgtaatgc	MSGTITFKGTVLGKEITGTVTGTPGTGTATADLGGEHFTGTISAGPLTVTLTNAKGDVITIKVDGTKVTVSGDVDGKKFEGEGTYTPGSDHIDLGVAEEIPGVGSGSHHWGSTHHHHHH
TMB_n12_S14_nres14_2	atactacggtctcaaggaAGCAAAGAGAAAGAATACGGCGCGGAACTGGGCCTGGGCTGGCGCGACAACAAATA	MGSSKEKEYGAELGLGWRDNKYYIYAG

	CTATATTTACGCGGGCACCTATAGCGAAGATGGTGA<br>AAAGCGCGACGAAGAAAAGAAGAAAGAAAGCCTG<br>GGCGTTGATCTGGGCCTGGGCCTGAAAGAAGAAC<br>GCAAATATTATGTGTATGTGGGCGCGTATGTGGAAT<br>TTGGCGAAAGCGATGATAAGGACAAAGAACTGTAC<br>GTCGGCAGCTATCTGAAACTGGGTCTGGATGATGA<br>ATGGGAAGCGAAACTGGGCCTGTATGCGGGCGGC<br>AAATACAAAGACAAAGATAAAGACTTCGAAATTTAC<br>GTGGGCGTGTATGGCGGTGCGGGTGCGGATAAAA<br>CCAAGAAACTGTATTACGAATTCGGCGTTTATACCG<br>GCCTGGGTTTTGATAGCAAGAAGGATAAAGAGAAA<br>GGCTATTTCGGCACCGGTCTGGGCGGCCAGTATAA<br>AGATAACCAGTACAAATATCGCGGCGATATTGAAGC<br>GGGCATTGGCTATGATGATTCCAAGAAAGATAGCG<br>CCTATGTGAAAATTTATGCGGGTTGGGATGATCGCA<br>ATAAATTCGAAGCGGGTCTGGGTAGCGGCTTTGCG<br>CAGAAGAAATATGATAAAGAATAATAGggttcccgagacc<br>gtaatgc	TYSEDGEKRDEEKK<br>KESLGVDLGLGLKE<br>ERKYYVYVGAYVEF<br>GESDDKDKELYVGS<br>YLKLGLDDEWEAKL<br>GLYAGGKYKDKDKD<br>FEIYVGVYGGAGAD<br>KTKKLYYEFGVYTG<br>LGFDSKKDKEKGYF<br>GTGLGGQYKDNQY<br>KYRGDIEAGIGYDD<br>SKKDSAYVKIYAGW<br>DDRNKFEAGLGSGF<br>AQKKYDKE
TMB_n12_S14_nres14_1	atactacggtctcaaggaAGCTATGAGAAAGAACTGGGCG<br>TCGAACTGGGCGCGGGCCTGCGCGATAACAAATT<br>CTATGTGTATATTGGCGCGTATAGCGAAGATGGCGA<br>TAAAGATGATGAAGAGAAGAAGAAGAAAAGCTACG<br>GTTTTGACGTTGGCGGCGGCGCGAAAGAAGAACG<br>CAAATTTTATTTCTATCTGGGTGTGTACGCGGAATAT<br>GGCGAAAGCGATGACAAAGATGAAGAACTGTATAC<br>CGGCCTGTATAGCAAAGCGGGCTTTGATGACGAAT<br>ATGAACTGAAAATCGGCCTGTACATCGGTCTGAAA<br>TATAAAGACAAGGACAAAGACTTCGAATTCTACGCA<br>GGCCTGTATGCGGGTACCGGTTATGATAAAACCAA<br>GAAATGGTATCTGGAAGTGGGCGTTTACGCGGGTC<br>TGGGCTATGATCGTAAGAAAGATAAAGAAAAGGGC<br>TATATCGGCGTGGGCGGTGGCTATCAGGGCAAAG<br>ATAACCAGTATAAATATCGTCTGGATGCGGAAAGCG<br>GCGCGGGTTGGGATGATAGCAAGAAAGAAAGCGC<br>GTACGTTAAAATTTATCTGGGCCTGGATGATCGCAA<br>CAAATGGGAAGGCGGCTTAGGTGTGGGCACCGCG<br>AAGAAAGATTATAAGAAAGAATAATAGggttcccgagacc<br>gtaatgc	MGSSYEKELGVELG<br>AGLRDNKFYVYIGA<br>YSEDGDKDDEEKKK<br>KSYGFDVGGGAKE<br>ERKFYFYLGVYAEY<br>GESDDKDEELYTGL<br>YSKAGFDDEYELKI<br>GLYIGLKYKDKDKDF<br>EFYAGLYAGTGYDK<br>TKKWYLEVGVYAGL<br>GYDRKKDKEKGYIG<br>VGGGYQGKDNQYK<br>YRLDAESGAGWDD<br>SKKESAYVKIYLGLD<br>DRNKWEGGLGVGT<br>AKKDYKKE
TMB_n12_S14_nres14_3	atactacggtctcaaggaCGTGAGAAAGAATTTGGCGCGG<br>GCCTGAAACTGAAAAGCAAAGAAGATAACAAATAT<br>GATGCGGGCAGCGAAGTGTATTTTGGCCATCGCG<br>ATGAGAAAGATAAACGCTATCTGTATGGCGGCGTG<br>GAATTTGAATATAGCGATGACAAGAAAGATTATGAA<br>ATTAAAGCGTATGTGGGTTATGGCTATAAAGAAAAC<br>AAACAGGAATATGGCCTGGATTTCTATTTTGGTTTT<br>GGTCTGCGTGATGATAAATTTACCTATGATGTGGGC<br>TTTGGCATTCAGAGCGGCTCCGAAGAAGATAAATA	MGSREKEFGAGLKL<br>KSKEDNKYDAGSEV<br>YFGHRDEKDKRYLY<br>GGVEFEYSDDKKDY<br>EIKAYVGYGYKENK<br>QEYGLDFYFGFGLR<br>DDKFTYDVGFGIQS<br>GSEEDKYKYGWGA<br>KLGSKYYDKKLSVY

	TAAATATGGTTGGGGCGCGAAACTGGGTAGCAAATATTACGATAAGAAACTGAGCGTGTATGTTGGCCTGTATGCGGAACTGGGCTATGAAGAAAATAAATACAAATATCGCTATGGCACCGGTACCGGCCTGGAAGCGAAAGATGATTGGTTTGATCTGTATATTGAAGTGGGCCTGGGCCTGGGTTATCAGGATGATAAGAACCAGGAATGGTACGCGGGCGGCAAACTGCGTGCGGGCGCGGAAGATAATAAATATAAAGGCTATACCGAAGTGAACATTGGCGGTGGCCATAAAGATAAAGAAGATTATTAATAGggttcccgagaccgtaatgc	VGLYAELGYEENKYKYRYGTGTGLEAKDDWFDLYIEVGLGLGYQDDKNQEWYAGGKLRAGAEDNKYKGYTEVNIGGGHKDKEDY
TMB_n12_S14_nres14_4	atactacggtctcaaggaCGCAAACAGGATAAACTGGGCACCCAGGTGGGCTTCGGCGCGCGCGATAAGAAATGGGAAGTGTATCTGGGCTTTTGGCTGGAACAGGGTAAAGAGAAGGATGACAAGAAAGATCGTGATAAATTTGGCATTGAACTGGGTGCAGGCGCGGATAGCAAGAATAAATATCGTATTGAATTGGGCCTGTGGATTGAATATGGCAAAGAAGATGATAGCGAGAAAGAACTGTATGCGGGCACCTATCTGAAAAGCGGTTGGAAAGATGAATACGAAGTGGAATTCGGCGTTTACGCGGGCGTGCGCTACCGCGATGATAAAGATGACTATAAAGTTGAAGCGGGTCTGTATAGCGGCGGTGGCTTCGATAAAGATAACAAATGGTATTTCAAACTGGGTAGCTATACCGGCGCGGGCTTTGAAAAGAAGAAAGACAAAGAAGAAGTGTACGCCGGTGGCGGCTTTGGCGGCGAACTGGAAAAGGACGAATATAAACTGAAAATTTATTTTAAAGTGGGTGCGGGCTACAAAGACGATAAGAAGGAACGCCTGTATATTGAAGCCTATGTGGGCTATGATTCTAAGAACAAATATGAATTAGGCGCCGGCCTGGGTTTAGAAAAGGCGGATGATTATAAAGAATAATAGggttcccgagaccgtaatgc	MGSRKQDKLGTQVGFGARDKKWEVYLGFWLEQGKEKDDKKDRDKFGIELGAGADSKNKYRIELGLWIEYGKEDDSEKELYAGTYLKSGWKDEYEVEFGVYAGVRYRDDKDDYKVEAGLYSGGGFDKDNKWYFKLGSYTGAGFEKKKDKEEVYAGGGFGGELEKDEYKLKIYFKVGAGYKDDKKERLYIEAYVGYDSKNKYELGAGLGLEKADDYKE
TMB_n12_S16_nres14_1	atactacggtctcaaggaGATAAAGGCTGGCGCGCCGAATTCGAATTTGAATGGTATCGCGATAACCGCTATAAAGCGGGCGTTGCGCTGGGCGTGAAACTGGGCAAGAAAGACGAAGATAAGAAAGGCTATTACGCTGAAGCGGGTGTGAAATTAGGCTTCAAAGATAACAAACTGTACAGCGGCCTGTATGCGCAGGTGGGCTACGAAAAGAGCGAAGACGAGAAACTGAAATATCGTCTGTATAGCTACTTTGGCTTCGGCTGGGATGATGATAAAGGTTATATTTATGCGGGCACCTATCTGGGCCTGCAGAACGAAGACAAGAAGGATAAGAAATATTATTACGGCGATACCGGCCTGGATGTGAAGCTGGATAAAGACAACAAATATGATGGCGGCGTTAAAACCGGCATTGGCGTGGGCTTTAAAATATCATATTGACGACAAAGACAGCGAATACGAACTGGGTTTTCGCCTGGGTCTGGATAAAGATGGCGAATTTAAAAGCGGCATTCGCTTTGGCGCGCGCTATGAAGATAAAAGCGAGTATAAATATGTGTATCTGGACGCCCAGGTGGATTATTTTGAAGATCGCAAAGC	MGSDKGWRAEFEFEWYRDNRYKAGVALGVKLGKKDEDKKGYYAEAGVKLGFKDNKLYSGLYAQVGYEKSEDEKLKYRLYSYFGFGWDDDKGYIYAGTYLGLQNEDKKDKKYYYGDTGLDVKLDKDNKYDGGVKTGIGVGFKYHIDDKDSEYELGFRLGLDKDGEFKSGIRFGARYEDKSEYKYVYLDAQVDYFEDRKAEAIRIKLGAGYDEKKKD

	GGAAGCGATTCGCATTAAACTGGGCGCGGGCTATGATGAAAAGAAGAAAGATTAATAGggttcccgagaccgtaatgc	
TMB_n12_S16_nres14_2	atactacggtctcaaggaGATGAAGGCTGGGAAGTGGCGGTGGATGTGGAATATAAAGATGATCAGAAACTGAAAGCCGGCTTTCGCATTGGCAGCGAATTTGGCAAAGAGGATGAAAAGAAGAAAGGCTACTATTTTAAAGTCGGCCTGAAAAGCGGCCTGGAAGATAACAAATGGTATGCGGGCGTGCAGAGCACCGGCGGCTATAAAAGCGCGGAAGACGAAAAGAAAGAATTTGAAATTCAGTTTTATCTGGGCGGCGGTGCGAAAGATAAAAGCTATTATTTCTACCTGGGCATTGAAGGCGGCTGGCGCGAAAAGGATGATAAAGATAAGAAGAAATACGAAGCGAAAGTGGGCTTAGAAGCCAAATTTGATAGCAGCAACAAATATCGCGGTGGCTTTTATGCAGGCCTGGGTCTGGGCCTGCAGAAACGCGATAGCGAACGTGATAGCGATTATGAAACCGGCATTAAAGCGGGTCTGAAATCCGATGGCAAACTGGAAGCCGGTATTTATACCGGCTTAAAATATGAGAAGAAGAAAGAATATGAATATCTGTATGCGAAAACCGAAGTGAAATATGCGGATAAACGCAAAGGCGAAAGCGTGCGCGTGGAACTGGGTTTAGGCTACAAGAAGAAGAAGAAAGATTAATAGggttcccgagaccgtaatgc	MGSDEGWEVAVDVEYKDDQKLKAGFRIGSEFGKEDEKKKGYYFKVGLKSGLEDNKWYAGVQSTGGYKSAEDEKKEFEIQFYLGGGAKDKSYYFYLGIEGGWREKDDKDKKKYEAKVGLEAKFDSSNKYRGGFYAGLGLGLQKRDSERDSDYETGIKAGLKSDGKLEAGIYTGLKYEKKKEYEYLYAKTEVKYADKRKGESVRVELGLGYKKKKKD
TMB_n12_S16_nres14_3	atactacggtctcaaggaAAGAAATATGGCTACAAATTTGGCGTTTATACCCGCTGGTACGATGATAACGAATATGAACTGGGTAGCGGCTTTCATCTGGTGTATAAAGATGAAAGCGACAAAGGCTATAAACTGGGCGTGGGTGCGAAATACGATAAAGATAAAGACAAACTCGAAGCGGGCGGCGATTTTCAGTGGTACAAAGAAAAGAAAGACGACGAAGAGAAATGGCTGTACGTGTATGCGAAACTGGATGAAAAGGATAAGAAAGATCTGGAAATTGGTACCGGCGCGGGCTTTGGCAAAGAAGACAAGAACAACGAAAAGTATTACGTCGGCGTCTATGGCGGCCTGGGTTATGAAGATGATAAAGGTTATCTGTATGCCGGCCTGTATGTGGGCGCGCGCTGGGATGCGGATCGCAAATATAAATTTGGCACCGGCGTGTATAGCGGCGCGTACCTGTACGAAGATAAGAAATACAGCGCGGAAGTGGGTCTGTATATTAAAGTGGGCTGGTATGACAAAGATAAATTCGAAATCGGCAGCAAATTCGGTGCGGGTCTGGATCGTAAGAACCGCTTTTATATTGAATTTGGCCTGGAACTGGGCTATGGTGACAACGATCTGCTGCGCAAAAGCTAATAGggttcccgagaccgtaatgc	MGSKKYGYKFGVYTRWYDDNEYELGSGFHLVYKDESDKGYKLGVGAKYDKDKDKLEAGGDFQWYKEKKDDEEKWLYVYAKLDEKDKKDLEIGTGAGFGKEDKNNEKYYVGVYGGLGYEDDKGYLYAGLYVGARWDADRKYKFGTGVYSGAYLYEDKKYSAEVGLYIKVGWYDKDKFEIGSKFGAGLDRKNRFYIEFGLELGYGDNDLLRKS
TMB_n14_S16_nres12_1	atactacggtctcaaggaAAAGAAGGCGAAATTTATAGCGGCGCGGATCTGGATAAAGATAAAAAATATGAAGAACTGCGCTTTGGCACCCGCTATGATGAAAGCCGCAGCAAAGATAAAGATGATGATTATCTGGGCGTGGATTTTGAGAGTCGCGCGAAAAATAAAGCGTATCGCGAAG	MGSKEGEIYSGADLDKDKKYEELRFGTRYDESRSKDKDDDYLGVDFESRAKNKAYREGGLGLKWGSKSK

	GCGGCCTGGGCCTGAAATGGGGCTCGAAAAGCAAAGATGATAAGGACGAATTTGGCGTGCGCCTGGAAGCGGGCTGGGATGAAAAACGCAAATATTATGTGGGCGCCAAAGTGTATGCGGGCTATCGCTACGAGGATAATTTTAAAAGCGATTTCGAAGGGGAATTACGCTTGGAAGACGATTATAAACTGAAAGTGGAGGCCAAAATTGGTGTGAAATTGAAAGACGACAAAGGTGAATACGAAATTGGCGCGGGCTTCGGCGGCTACCGTGATGACGACAAAAAATTAAAATGGAAACTGGATATTTATGCAGGCATTAAATTTGACAAGGATAAATTTAGCAGCGGGGCGTGGGCGAGCGTGGAATATGATAGCAGCAATAAGAGTGATAAACTGAGCGCCGGCACCGAACTGGGGCTGCGCGATAAGGACAAAGAGCTGTATTTAGGCACCCAGCTGGACAAGGATCGCGATGACAAGGAAGAAAAAGTGCGCGTGTATGGCCGCGTTAAGAAACGCAAAAAAGATGATAAGGATGACAAATAGTAAggttcccgagaccgtaatgc	DDKDEFGVRLEAGWDEKRKYYVGAKVYAGYRYEDNFKSDFEGELRLEDDYKLKVEAKIGVKLKDDKGEYEIGAGFGGYRDDDKKLKWKLDIYAGIKFDKDKFSSGAWASVEYDSSNKSDKLSAGTELGLRDKDKELYLGTQLDKDRDDKEEKVRVYGRVKKRKKDDKDDK
TMB_n14_S16_nres12_2	atactacggtctcaaggaAAAGAAGGCGAATTTTATGCGGGCATTGATTATGATAAAGATAAAAAACTGGAAGAATTTCGCAGCGGCCTGCGCTATGATGAAAGCAAAGACAAGGACAAAGATGATGATTATTGGGGCGTGGATGTGGAAGTGCGCGCGAAAAATAAATTCTATCGCGAAATTGGCTCGGGCGTGAAATTTGGCAAAAAAAGTAAGGATGATAAGGAGGAATATGGCGCGCGCGTGGAACTGGGCTATGACGAAAAACGCAAATGGTATGCAGGCGTCAAAAGCTATCTGGGGTATCGCTACGAAGATAATTTTAAAATTGATCTGGAGGTGGAAGCGCGTTACGAGGACGATCTGAAAATTAAAACCGAAGTGAAAACCGGCCTGAAATATAAAGACGACAAAGGTGAACTGGAAGGCGGCTTTGGCCTGGGCGTGTATCGTGATGACGATAAGAAAGCCAAATGGAAGTTGGACGTGTACGCCGGCGGCAAATATGACAAAGATAAACTGAGCTTAGGCACCTGGGCGAGCGCGGAATTTGATAGCAGCAATAAGCTGGATAAGTTTAGCTCTGGCGCCGAGCTGGGCTTACGCGATAAGGATAAGGAAGCGTACGCGGGTCTGCAGATTGACAAAGATCGCGATGACAAGGAAGAAAAGCTGCGCCTGTATTTTCGCTTTAAGAAACGTAAAAAAGACGATAAGGATGACAAATAGTAAggttcccgagaccgtaatgc	MGSKEGEFYAGIDYDKDKKLEEFRSGLRYDESKDKDKDDDYWGVDVEVRAKNKFYREIGSGVKFGKKSKDDKEEYGARVELGYDEKRKWYAGVKSYLGYRYEDNFKIDLEVEARYEDDLKIKTEVKTGLKYKDDKGELEGGFGLGVYRDDDKKAKWKLDVYAGGKYDKDKLSLGTWASAEFDSSNKLDKFSSGAELGLRDKDKEAYAGLQIDKDRDDKEEKLRLYFRFKKRKKDDKDDK
TMB_n14_S16_nres12_3	atactacggtctcaaggaGAACGCTTTGAAGTGTGGGTGGGCCTGAGCCAGAGCAGCGATGATAAATATCGCGATGGCAAACTGGGCACCCGCTTGAGCGAAGAAGAGGATAAAAAAGATCAGAAACGCCAGGGCGGCATTGAGGTGAAAGCGAAATATAAAGATGATTATTATGAAGAAGCGGGCCTGGGTCTGTATTGGGGCGAAGCGGACAAAAGCAAAAAGAAATGGCTGGGGCTGAAAGTGGATGCGGGCTGGGATAAAGAAAATAAAGCGTATTTTG	MGSERFEVWVGLSQSSDDKYRDGKLGTRLSEEEDKKDQKRQGGIEVKAKYKDDYYEEAGLGLYWGEADKSKKKWLGLKVDAGWDKENKAYFGVDAEFGFQDDDYLRTE

	GCGTGGACGCGGAATTTGGCTTTCAGGATGATGAT TATCTGCGCACCGAATTTAAAATTAGCTTGAAATTC AAGGATGATGCGTATACCCAGGCGGAAGTGCGCG TGGGCATCGAATATAAGGACAAAGATGGCGATCTG GAAGCAGGCAGCGGGATTGGTGTGCGCAAAGATA AGGACCAGAAATATGAATGGTATAGCTATCTGTATG CCGGCGGCCGCTATAAAAAGGATCGCTTTCGCAGT GGCATTTATGCGCAGCTGGATTATGATAGCAGCTAT AAGAGCGAAAAACTGCGCTTTGGCCTGGAGTTTG GTTTGGATAATGATAAATATGAACTGGATGCTGGCG CGGAAAGCCAGTATCAGCGCGATCGCAAGAAGGA AGAAGTGGAAGTGTATCTGAAATTTCGCGAGGAAA AAGACAAACAGGACGACGATAAATAGTAAggttcccga gaccgtaatgc	FKISLKFKDDAYTQA EVRVGIEYKDKDGD LEAGSGIGVRKDKD QKYEWYSYLYAGG RYKKDRFRSGIYAQ LDYDSSYKSEKLRF GLEFGLDNDKYELD AGAESQYQRDRKK EEVEVYLKFREEKD KQDDDK
TMB_n14_S16_nres12_4	atactacggtctcaaggaGATGATCTGCGCCTGTGGGCGG GCACCAATAAAGATAAGGATGATAAATATGAAGAAA GCTATCTGGGCTTTGAATTTCGCAAAAGCGATGAT GATAGCGATAAATATAAAAAATGGGGCGTGTATGGC TACCTGAAATACAAGGATGATTGGTTTAAAGAAGCG GGCGTGGGTGTGGAATTTGGCGATGAAAGCAAAG ACAAATCGTATTGGCTGGGCAGCCGCATTGAACTG GGCCTGGATGAGGAACGCAAATATTATTTAGGCGC GAAAATTTATGGCGGCTATCGCAAGGACGACAATTA TGAGAGCGAAGTGGAAGTTGAAGTGAAAGGCTATT CGGATTATTACTTTGCGGCGGAGGTGAAATTGGGT GCGAAACTGAAAGACGATAAAGGCGATCTGCAGTT TGGCTTTGGCGCGGGGACCGAAGATGACAAGGAC AAAAAATGGCGCTTTTATATTAAATTTGAAAGCGGC CTGAAGGCGCGCCGCGATAAGTACGACTTGGGGC TGTATGCGGATTTTAAAGCGGATAGCAAAAATGAAA AAAAAGATGCGAAAATTGGCCTGAGCACCGGCTAT AAACGCGATAAAAAGAAGGCCGAGGTGGGCGCGG AGGTGGATCAGAGCAGCGACGACAAAGATGAGAA ACTGCGCGTTGAATTGAAGATTGAAGATAAGGAAA AGAAAGATAAAGACGATAAATAGTAAggttcccgagaccg taatgc	MGSDDLRLWAGTN KDKDDKYEESYLGF EFRKSDDDSDKYKK WGVYGYLKYKDDW FKEAGVGVEFGDES KDKSYWLGSRIELG LDEERKYYLGAKIY GGYRKDDNYESEV EVEVKGYSDYYFAA EVKLGAKLKDDKGD LQFGFGAGTEDDKD KKWRFYIKFESGLK ARRDKYDLGLYADF KADSKNEKKDAKIG LSTGYKRDKKKAEV GAEVDQSSDDKDE KLRVELKIEDKEKKD KDDK
TMB_n14_S16_nres12_5	atactacggtctcaaggaAAAGATCTGCAGGTGTATGTGG GCGGCGATTATGATGAAGATGAAAAATTTAAAAGCT TTTATGCGGGCGCGAAATGGGAAGAAGAGGAGGA TAAAAGCAAAGACAAAGAACGCTTTGGCGCAAAAG TGGATGTGGAACTGAAAGATGATCTGTATCGCAGC CTGGGCGCGGGCTTTGAAGGCGGCCGCAAAAAG AAGGACGACGAATATGAACTGGGCGTGAAAATTGA TGCCGGCTGGGATGAAAAACGCGACTATTATTTTG GCGTGGAAACCGAATTAGGCCTGCAGGATAAGGAT TACTTTTATATTAAATTTAAACTGCGCGGCGAATATA AAGACAAATATTATCTGAAATTTGATGCGGAAGTGG	MGSKDLQVYVGGD YDEDEKFKSFYAGA KWEEEEDKSKDKE RFGAKVDVELKDDL YRSLGAGFEGGRK KKDDEYELGVKIDA GWDEKRDYYFGVE TELGLQDKDYFYIKF KLRGEYKDKYYLKF DAEVGSKYKDKDG RFEAGSGLGVRKDK

	GCAGCAAATATAAGGATAAGGATGGCCGCTTTGAAGCGGGTAGCGGCCTGGGGGTGCGCAAAGACAAAGATGATGATGCGAAATACGAGATTTATCTGCGCTTGGGCACCGAAGCGAAAAAAGATCGCTATGATACCGGCCTGTATATTCAGAGCGATTATGATAGCAGCAATAAAAAAAAACGTCTGCGCGCCGGCGCGTATATTGGCTGGGATTACGATAAAGACCGTAGCCGCGCAGGCCTGGATGGCGAGTACGAAAGCGATAATAAGAAAAAGAAATTAAAAGTGTGGCTGGAGGTGGAAAAGAAAAAAAAAGATGATAACAAAAAAGATTAGTAAggttcccgagaccgtaatgc	DDDAKYEIYLRLGTEAKKDRYDTGLYIQSDYDSSNKKKRLRAGAYIGWDYDKDRSRAGLDGEYESDNKKKKLKVWLEVEKKKKDDNKKD
TMB_n16_S20_nres12_2	atactacggtctcaaggaAGCAAATTTAAGCTGGATACCGATCTGGAAATTAAATTCGATGATAGCGCGAAATGGAGCGCGGATGCGAAAGTGAGCGCGAAGAACAGCGACGATCTGGATGCGAGCCTGAGCCCGCCGAAATATAGCGATGATAAATACGTGCTGAAGCTGAAAATTACCGTTCAGTGGCCGACCAGCGGCGCCAAAGGCGATGTGGATAGCAGCTATGATTCGGATGATAACAAAGCGAGCTATAGCATTGCGATTACCCTGCCGCTGTGGGATAGCAACGATGTTAAAGGCACCAGCAGCGGCAGCTATTCTGATAGCAAATACGATTATGAAGTTACCGCGACCGGCAAAGATCCGAGCGGCGTGGATATTGATTTTACCGGCCGCTTTAGTGATGATGACGATTTTGATTATACCCTGAGCACCAAAGTGCAGTATCCGAAAGATAACCTGACCCCGACCTTTGATGCGACCCTGCAGGATGGCAGCCTGAAAGCGACCTTTCTGCTGAAACTGGATACCCCGGATTGGGGTGGTCCGAAACTGAGCGTGAGCGTGCAGAGCGATCTGGACAGCAACGATAACTATAAACTGACCCTGACCGCGGATGTGAAATTTCCGGGCGCGCTGGTTAGCGTGTCCACCAAATATGATAGCAACACCGATAAACTGAGCCTGGATGCCAAACTGACCGCGAGCAGCCTGGATTAATAGggttcccgagaccgtaatgc	MGSSKFKLDTDLEIKFDDSAKWSADAKVSAKNSDDLDASLSPPKYSDDKYVLKLKITVQWPTSGAKGDVDSSYDSDDNKASYSIAITLPLWDSNDVKGTSSGSYSDSKYDYEVTATGKDPSGVDIDFTGRFSDDDDFDYTLSTKVQYPKDNLTPTFDATLQDGSLKATFLLKLDTPDWGGPKLSVSVQSDLDSNDNYKLTLTADVKFPGALVSVSTKYDSNTDKLSLDAKLTASSLD
TMB_n16_S20_nres12_3	atactacggtctcaaggaAACGAAAAGATTAAAGTCGATAGCGGCCTGAAACTGGATGATGGCGATGCGAAATGGTGGACCAAAGTGAAACTGAAAACCAAGAAAGATGACGACTACGACGTGGAATCCAAAATTGATTTTGACGATGGCAAATATCATCCGGATATTAGCGTGACCGTGTATGATCCGACCGATCCGAACGATTATCTGACCGGCACCGTGAGCTTAGATGATGATGATAACGATCCGCCGAAAGTGACCTTTAGCGCGAAAGCGAAATACGGCCCGTTTGATCTGGAATTTAGCGTCACCCTGTTTCTGGATACCAACAAGAAGAAGAAAGTTCTGCTGAACGTGAAAGGCGATGATAGCGACGGCAAAAGCGCGCTGCTGAAAGCGGATCTGGATGACGATACCAATACCTATACCGCCGATCTGACCTTTTCGGGCAGCTTTCCGGGCTATAAATTTACCCTGACCAGCCAGTGGAACTATGATAGCAGCTATAAAGCGAACCTGAACAGCCTGGC	MGSNEKIKVDSGLKLDDGDAKWWTKVKLKTKKDDDYDVESKIDFDDGKYHPDISVTVYDPTDPNDYLTGTVSLDDDDNDPPKVTFSAKAKYGPFDLEFSVTLFLDTNKKKKVLLNVKGDDSDGKSALLKADLDDDTNTYTADLTFSGSFPGYKFTLTSQWNYDSSYKANLNSLAQLDFPGIKGTTKATWDSDTDDAKPDPPSLSLDYPDASA

	GCAGCTGGATTTTCCGGGCATCAAAGGCACCACC<br>AAAGCGACCTGGGATAGCGATACCGATGATGCCAA<br>ACCGGACCCGCCGAGCCTGTCTTTAGATTATCCGG<br>ATGCGAGCGCGTTTATTAAAGTGAGCCTGGATAAA<br>GATAGCGATAGCTACAAATGGGAAGCGCCGGATCT<br>GAGCCTGGAAGTGGATTAATAGggttcccgagaccgtaatg<br>c	FIKVSLDKDSDSYK<br>WEAPDLSLEVD
TMB_n16_S20_nres12_1	atactacggtctcaaggaAGCAGCGATCCGAAACTGAAAG<br>CGGGCCCGAAACTGAGCGACGGCAAATGGCGCTT<br>TTATACCAGCGCGACCGTGGATCTGAAAAGCGATG<br>ATAGCTATAAAAGCTCGGTGACCCTGGATCTGCGC<br>GATGGCAAATACGATATTAAACTGACCCTGAGCGT<br>GCTGGATCCGACTGATCCGACCGATAGCGCGGAA<br>CTGAGCGGTCAGTATGATCTGGATGATAACGATCC<br>GCCGAAAAGCACCATTAGCGCGAAATTTAAATTTG<br>GCCCGTTTGATGGCCAGCTGAGCCTGACCTTTTG<br>GCTGGATACCAACAGCCGCAAAAGCAGCGAACTG<br>AGCGTGAAAGTGGATGATTCGGATGGCCGTCGTG<br>CGCAGCTGAGCGCGCTGTTAGATGATAGCACCAAC<br>TCTTATACCTTTGTGGGCACCATCGATACCAAACTG<br>CCGGGCTGGGATGGCAGCACCACCATTAAAGCGG<br>ATTGGGATAGCAGCGCCAAATATCAGCTGAGCGTG<br>ACCGTGACCAGCGAATTTCCGGGCCTGAAAGCGA<br>GCGCAAGCGTGAGCTATGACAGCAACACCGACAG<br>CTATCAGCTGACCGGCCCGGATATCAAAGTTGATTA<br>TCCGAACGGCGATGCGAGCGCGTCGCTGACCTAT<br>GATAACACCAACGATAAATATGATGCGAAACCGCC<br>GGATGTGCGCGTTAAAGTGGATTAATAGggttcccgag<br>accgtaatgc	MGSSSDPKLKAGPK<br>LSDGKWRFYTSATV<br>DLKSDDSYKSSVTL<br>DLRDGKYDIKLTLSV<br>LDPTDPTDSAELSG<br>QYDLDDNDPPKSTI<br>SAKFKFGPFDGQLS<br>LTFWLDTNSRKSSE<br>LSVKVDDSDGRRA<br>QLSALLDDSTNSYT<br>FVGTIDTKLPGWDG<br>STTIKADWDSSAKY<br>QLSVTVTSEFPGLK<br>ASASVSYDSNTDSY<br>QLTGPDIKVDYPNG<br>DASASLTYDNTNDK<br>YDAKPPDVRVKVD
TMB_n16_S20_nres12_4	atactacggtctcaaggaAGCCTGCCGGAAAACGCGGGC<br>ATTAGCTATAAATGGGATCCGGATGATTTTAGCTTTG<br>AAAGCAAACTGACCCTGAAAGACCCGGATAAAGAT<br>AGCCGCGAAGCGAGCCTGGATCTGAGCGTGAAAG<br>CCGATGATAACTTCAAAAGCGATTACGACCTGAAA<br>GTGAAACTGTCGCTGCCGGGCGCGGATGTGGATG<br>TGCTGGTGACCTATAACGATAGCAGCGATCTGTCG<br>TATAAATATGATGTTACCGTGCAGTTTCGCCTGCCG<br>ACCTTTGATGTGACCGCGAGCAGCAGCCTGGATAA<br>TGATGATCCGACCAGCAACAAATATAGCAGCACCG<br>TTAAACTGGCGAAAGATCCGAGCGCGGATATTGAT<br>GGCACCGTGACCTGGGATCCGACCTCCGCGAAAT<br>TTACCCTGACCCTGCGCCTGAAACTGGATACCAAC<br>GATCTGGATCTGAAAGCGGATTATACCTGGAGCAG<br>CGATGATCCGCCGAAATATACCACCAGCGTTAAAG<br>CAGATTTTAAATATCCGAGCCATAAAGGCGATATCG<br>TTGCGAAATACGATCTGAAGAACGGCGCGTATAAG<br>AAAGCGGACGGCACCACCAAACCGGATTATCCGG	MGSSLPENAGISYK<br>WDPDDFSFESKLTL<br>KDPDKDSREASLDL<br>SVKADDNFKSDYDL<br>KVKLSLPGADVDVL<br>VTYNDSSDLSYKYD<br>VTVQFRLPTFDVTA<br>SSSLDNDDPTSNKY<br>SSTVKLAKDPSADID<br>GTVTWDPTSAKFTL<br>TLRLKLDTNDLDLKA<br>DYTWSSDDPPKYTT<br>SVKADFKYPSHKGD<br>IVAKYDLKNGAYKKA<br>DGTTKPDYPGTSPS<br>ISWDLDSDTDKLSF<br>DASPPDGKLD

	GCACCAGCCCGAGCATTAGCTGGGATCTGGATAG CGATACCGATAAACTGAGCTTCGATGCGAGCCCGC CGGATGGCAAACTGGATTAATAGggttcccgagaccgtaat gc	
TMB_n16_S16_nres14_1	atactacggtctcaaggaGATTATAAAGCGGATGCGCGTAC CCGCGCGGCGCTGGAAAAAGATAAAGACAATAAAG AAGAAAAACTGGAAATTCAGGTGGATTTTGAAAGC AAATGGTGGGATCGCAAAGATGAAGAATTTCAGGT TGAACTGAGCAGCAAATATGAAAAACGCAAAAAGG ATTACAAGGACGATGATAAATACGATGTGGCGCTGA AAGCGCGCGTGAAATTGGATAAGGATCAGTCGAGC AGCGAAGTGGAATCGAAATATACCGATAAGGATAG CAAAAAAGACTATGATGTGAGCCTGAAACTGGCGA TTAAACGCAAATTTCTGGACAAGGATGATTACGAGA GCGAAGGCAGCGCGGAAGGCCAGTTTAAATTTGA TAAGGATAAACGCGAAAAAGATGCGAAACTGCGTG TTAAGTTAGACTTTCGCTGGAAGAAGGACAAGTAC GAAAGTAAATTGCGCATTCGCGCCGAATACGAGGA AAAAAAGGAGAAAAAAGATAAGCGCAAACAGTTTG AAGCGGAAGCGCAGGCGGATTATGATGAATCGGAT GCAAAAGTGCAGATTAAAACCAATCTGGCGTTGGA TGAGGAGGAAGACAAAGATAAAAAGAAAAAGAAAG AATATCGCCTGGAAAGCAGCCTGGCGGTGCAGTT CAAATTTAGCCAGCTGGATGGCCGCATTCAGTTTA GCGCGAGCTTTAAAACGAAAAAGGACGATCGTAAG GAGGACGAGAGTGAATTCCAGGCGAGTTTTGAAG TGTATTATAATAAGGATAAAGTGCGCTCGAAAGTGG ATACCAAAAAAAAAGAGAAGGATGTGGATGAAGAT AAGAAGAAACGTGAAAGCTGGCGCTTGGAGTTGG AACTGCAGTTGGAGGATAAGAAAAAAGACGATTAG TAAggttcccgagaccgtaatgc	MGSDYKADARTRA ALEKDKDNKEEKLEI QVDFESKWWDRKD EEFQVELSSKYEKR KKDYKDDDKYDVAL KARVKLDKDQSSSE VESKYTDKDSKKDY DVSLKLAIKRKFLDK DDYESEGSAEGQF KFDKDKREKDAKLR VKLDFRWKKDKYES KLRIRAEYEEKKEKK DKRKQFEAEAQADY DESDAKVQIKTNLAL DEEEDKDKKKKKEY RLESSLAVQFKFSQ LDGRIQFSASFKTKK DDRKEDESEFQASF EVYYNKDKVRSKVD TKKKEKDVDEDKKK RESWRLELELQLED KKKDD

Table S3. Crystallography data collection and refinement statistics.

	n6_S8_nres10_D2
**Resolution range**	38.87 - 2.03 (2.08 - 2.03)
**Space group**	P 32 2 1
**Unit cell**	74.19 74.19 97.635 90 90 120
**Unique reflections**	20611 (1442)
**Multiplicity**	13.5 (13.0)
**Completeness (%)**	99.95 (99.72)
**Mean I/sigma(I)**	14.27 (1.01)
**Wilson B-factor**	50.84
**R-merge**	0.1028 (2.213)
**R-pim**	0.02934 (0.6344)
**CC1/2**	0.998 (0.644)
**Reflections used in refinement**	20611 (1442)
**Reflections used for R-free**	2003 (138)
**R-work**	0.2197 (0.3306)
**R-free**	0.2652 (0.3845)
**Number of non-hydrogen atoms**	2165
**macromolecules**	2070
**ligands**	38
**solvent**	57
**Protein residues**	270
**RMS(bonds)**	0.002
**RMS(angles)**	0.51
**Ramachandran favored (%)**	98.86
**Ramachandran allowed (%)**	1.14
**Ramachandran outliers (%)**	0.00
**Rotamer outliers (%)**	0.00
**Clashscore**	2.60
**Average B-factor**	62.16
**macromolecules**	61.96
**ligands**	75.57
**solvent**	60.29

Statistics for the highest-resolution shell are shown in parentheses.

SI References

1. J. Wang, *et al.*, Scaffolding protein functional sites using deep learning. *Science* **377**, 387–394 (2022).

2. M. Baek, *et al.*, Accurate prediction of protein structures and interactions using a three-track neural network. *Science* **373**, 871–876 (2021).

3. J. L. Watson, *et al.*, De novo design of protein structure and function with RFdiffusion. *Nature* **620**, 1089–1100 (2023).

4. M. Baek, *et al.*, Efficient and accurate prediction of protein structure using RoseTTAFold2. [Preprint] (2023). Available at: https://www.biorxiv.org/content/10.1101/2023.05.24.542179v1 [Accessed 12 January 2024].

5. J. Jumper, *et al.*, Highly accurate protein structure prediction with AlphaFold. *Nature* **596**, 583–589 (2021).

6. I. Loshchilov, F. Hutter, Decoupled Weight Decay Regularization. *arXiv.org* (2017). Available at: https://arxiv.org/abs/1711.05101v3 [Accessed 12 January 2024].

7. A. Paszke, *et al.*, PyTorch: An Imperative Style, High-Performance Deep Learning Library. *arXiv.org* (2019). Available at: https://arxiv.org/abs/1912.01703v1 [Accessed 12 January 2024].

8. J. Dauparas, *et al.*, Robust deep learning-based protein sequence design using ProteinMPNN. *Science* **378**, 49–56 (2022).

9. K. E. Wu, *et al.*, Protein structure generation via folding diffusion. [Preprint] (2022). Available at: https://arxiv.org/abs/2209.15611v1 [Accessed 18 November 2022].

10. J. Yim, *et al.*, SE(3) diffusion model with application to protein backbone generation. *arXiv.org* (2023). Available at: https://arxiv.org/abs/2302.02277v2 [Accessed 11 April 2023].
